# Supplementary material for: Function and Characterization Analysis of BodoOBP8 from Bradysia odoriphaga (Diptera: Sciaridae) in the Recognition of Plant Volatiles and Sex Pheromones
Source: Insects. 2021 Sep 28;12(10):879. doi: 10.3390/insects12100879 (PMC8539145; doi:10.3390/insects12100879)
Supplement: Supplementary file 1 [file insects-12-00879-s001.zip › Figure legend.pdf]

Figure legend

Figure S1. The phylogenetic analysis of BodoOBP8. A neighbor-joining tree was constructed based on the amino acid sequences of the odorant-binding proteins (OBPs) for *Bradysia odoriphaga* and other insects. BodoOBP8 is indicated by a red colour. Bootstrap values were calculated with 1000 replications, and those larger than 50% are marked on the nodes. The protein names and sequences of the 228 OBPs used in this analysis are listed in Supplementary file 1. Bodo= *Bradysia odoriphaga*; Agam= *Anopheles gambia*e; Dmel= *Drosophila melanogaster*; Aaeg= *Aedes aegypti.*

Figure S2. SDS-PAGE analysis of the recombinant BodoOBP8. Line 1: crude bacterial extracts before induction by IPTG; Line 2: crude bacterial extracts after induction by IPTG; Line 3: inclusion body of induced BodoOBP8; Line 4: supernatant of induced BodoOBP8; Line 5: purified BodoOBP8 with His-tag; Line 6: final purified protein digested by enterokinase.
