# Supplementary material for: Function and Characterization Analysis of BodoOBP8 from Bradysia odoriphaga (Diptera: Sciaridae) in the Recognition of Plant Volatiles and Sex Pheromones
Source: Insects. 2021 Sep 28;12(10):879. doi: 10.3390/insects12100879 (PMC8539145; doi:10.3390/insects12100879)
Supplement: Supplementary file 1 [file insects-12-00879-s001.zip › Supplement files.pdf]

Supplementary file 1

>BodoOBP8

MKFFIAISLALVSTCFAADLRRDEKWPPKALLEYLEPIRISCMGKTGVTAEAIKEFSDGELHDDPKLKCY
MNCVFNEAKVVDDKGDVHFEKIDTHIAQLDDEIRHIAENFLANCRSIKGDDPCERAFSVHKCWKLH
DPKHYFLP

>DmelOBP8a

MAHCMYILLLLLLLVVELTPPAIPVPMRSSPQSLALLRARDQCGRELTAAQRLQLDRMQFEDAAHVRH
YLHCFWSRLQLWLDETGFQAQRIVQSFGGERRLNVEQALPAINGCNAKTSSRGSGAQTVVDWCFRAF
VCVLATPVGEWYKRHMSDVINGNA

>DmelOBP18a

MKVVCSIAVLWICLITMWQSAGRVNAEGCLKHHNLTSAQVQAVAPSTPVADVPVAVKCYSRCLIQDY
FGDDGKIDLQKVGKRGSQEDHVILSQCKQQFDGVTNLDTCDYPYLILQCYFKGKQSGTIAS

>DmelOBP19a

MKFHLLLVCVAISLGPIPQSEAGVTEEQMWSAGKLMRDVCLPKYPKVSVEVADNIRNGDIPNSKDTN
CYINCILEMMQAIKKGKFQLESTLKQMDIMLPDSYKDEYRKGINLCKDSTVGLKNAPNCDPAHALLS
CLKNNIKVFVFP

>DmelOBP19b

MTNLLLAVACAAVLMGSATADEEEGSMTVDEVVELIEPFGDACTPKPSRENIVEMVLNKEDAKHETK
CFRHCMLEQFELMPEDQLQYNEDKTVDMINMMFPDREDDGRRIVKTCNEELKAEQDKCEAAHGIA
MCMLREMRSSGFKIPEIKE

>DmelOBP19c

MKPSTPVAAIPLMTIVVAVLLQTHCVRGQTQAFDLAKLLPKTGTEPIWAVIDRNLPQVQELVTAARME
CIQKLQLPRDQRPLGKVTNPSEKEKCLVECVLKKIKLMDADNKLNVGQVEKLTSLVTQDNKMAIAVS
SSMAQACSRGISSKNPCEVAHLFNQCISRQLERNNVKLVW

>DmelOBP19d

MSHLVHLTVLLLVGILCLGATSAKPHEEINRDHLLELANECKAETGATDEDVEQLMSHDLPERHEAK
CLRACVMKKLQIMDESGKLNKEHAIELVKVMSKHDAEKEDAPAEVVAKCEAIETPEDHCDAAFAYEE
CIYEQMREHGLELEEH

>DmelOBP28a

MQSTPIILVAIVLLGAALVRAFDEKEALAKLMESAESCMPEVGATDADLQEMVKKQPASTYAGKCLRA
CVMKNIGILDANGKLDTEAGHEKAKQYTGNDPAKLKIALDIGETCAAITVPDDHCEAAEAYGTCFRG
EAKKHGLL

>DmelOBP44a

MKNAVAILLCALLGLASASDYKLRTAEDLQSARKECAASSKVTEALIAKYKTFDYPDDDITRNYIQCIF
VKFDLFDEAKGFKVENLVAQLGQGKEDKAALKADIEKCADKNEQKSPANEWAFRGFKCFLGKNLPL
VQAAVQKN

>DmelOBP46a

MCSQLFAFLLLLLTAFVTGRSTPPALDEDCELNSVDTMHDFCCDLHDESPQFSDCQMEWHEKIPYETD
EEEQTYMFCTAECSFNSTNFLGRDRRSLNLNEVKEHLESDLVNDADIKLLYDTYVKCDKHALSLMPH
KGVKQLSKRLSRLGCHPYPGLVLECVANEMILHCPTKRFRQTAQCEETRNHLKQCMQYLKYKS

>DmelOBP47a

MNRVLVLLLVLKMFALSESRFAKININLGLTVADESPKTITEEMIRLCGDQTDISLRELNKLQREDFSDPS
ESVQCFTHCLYEQMGLMHDGVFVERDLFGLLSDVSNTDYWPERQCHAIRGNNKCETAYRIHQCQQQ
LKQQQQNLLATKEVEVTTTPAGSDETKP

>DmelOBP47b
MSPSQLLVIFASLALNTRLVFGQATIDCQRPPQLVDPALCCKDGGRDQVAEQCAQRILGTANGQKAG
GPPSLDTAACLAECILTSSKYIDEPQKLNLANIRSDLSAKFSNDTLYVETMTMAFSKCEPQSQRRLAMI
MQQQQQVQQQKTQQQQPRCSPFSAIVLGCTYMEYFKNCPDHRWTPNAQCTLAKAYVTQCGLGA
>DmelOBP49a
MLSKSQLLLLVVGFCLNAAVSADVDCSKRPSFVNPKTCCPMPDFVTAELKQKCIKFDMTPPPPPDGEA
SGSFESKRRHHHPHPPPCFFSCIFNETGIYQNRKLDEAKLNAYLQEVFEDSSDLQTTATQAFTTCATKV
ADFEANLPPRPAPSPPPGFPMCPHDAGHLMGCVFRNMMKNCPDSIRNDSQQCTDMKEFFTKCKPPR
GPPPSAEDM
>DmelOBP50a
MRTGRILVALIFLGLIIPFRAAKCRAAPKSVQNVHVCCSAPLPNWGVFNRECHKSAIQASVSINRISKSK
VNLANFLIKCRLDCDFNASSVLQGNRLIQAKVRPMLERAFSNEPTIDAYESNFAKCSTVVRSKYQELSP
LSRQSDACDRHALFYSLCAYARLIFTCPDKMWQRNNRMCQEAKAYAKKCPWPALKMFMRNT
>DmelOBP50b
MSSVLHLLGFLWLPLLVYSVSNDMGGLQKCTELLNTHKLVYCCGKSFLDKFPFVGSNCTPFWDDYGP
CRYECLYRHWDLLDQDNKIKKPELYLMITSLYSPLNGYDKYGAAFKAAHETCEALGSRHADFLLLYSN
QVADKMGMASSTCLPYAMLHAQCTMVYLTANCPRENWIDDPKCNSLQKLLSSCTKKLDEKTNALK
GKDEELTDNGCGHIDSEGSNLLMACFLTLMIAKFISDH
>DmelOBP50c
MARHIALLICSLLAMAGCDPIDVDCTRRQDFNIVKDCCVYPTFRFDQFKSQCGKYMPVGAPRISPCLY
ECIFNKTNTVVDGAIHPDNARLMLEKLFGNQDFEEAYFNGLMGCSDSVQEMISNRRSRPQRKTEQCS
PFSLFYGICAQRYVFNHCPSSSWSGTESCEMARLQNMNCSKPSRGSSHRL
>DmelOBP50d
MLHKLTWVLIFIPAFRAADPICSQRPDVTALRNCCKLPNLDFSSFNSKCSQYLVNGVHISPCSFECIFRA
ANALNGTHLVMENIEKMMKTILGSDEFVHVYLDGFRSCGNQEKVLIKAMKRRRVPITGKCGSMAIM
YGLCAHRYVVYRNCPESVWSKSATCNEAREYSIRCDDM
>DmelOBP50e
MHKYIICFGFLLIILECSLASFNCSAPPNFNNFDINTCCRTPELDMGDVPQKCHKYVSGLKSANSKYPS
YAHLCYPDCIYRETGAMVNGKIKVNRVKQYLEEHVHRRDQEIVSHIVQSFESCLSNVKGHMKSLNIES
YKVLPHGCSPFAGIIYSCVNAETFLNCPQQMWKNEKPCNLAKQFAEQCNPLPHVPLPSS
>DmelOBP51a
MKVFIGLVLLLAVTTLSSALFESEANECAKKLGITPDYFENFPHSSRVKCFYHCQMEKLEIIANGVVTPF
DLKVLNISPESYDKYGVKVKPCLKLSHRDKCELGYLVFQCLKREFNL
>DmelOBP56a
MNSYFVIALSALFVTLAVGSSLNLSDEQKDLAKQHREQCAEEVKLTEEEKAKVNAKDFNNPTENIKCF
ANCFFEKVGTLKDGELQESVVLEKLGALIGEEKTKAALEKCRTIKGENKCDTASKLYDCFESFKPAPEA
KA
>DmelOBP56b
MKLIYLLVVFLIFALSELVAGQSAAELAAYKQIQQACIKELNIAASDANLLTTDKEVANPSESVKCYHSC
VYKKLGLLGDDGKPNTDKIVKLAQIRFSSLPVDKLKSLLTSCGTTKSAATCDFVYNYEKCVVKGISA
>DmelOBP56c
MYFRASLMALLCLTLSEFVSKAWVMFFIFYISFTRSLSVSLNMSMTRTLVPDPPNGTENKLSQEMLRAC
MRRTEISMSQLKLFHMSLMNSDYNNDNDIAPTPVQSIGDVNNLGDLDFNGNSQMPYLDLKHNEPLQ
CFVSCLYETLDLDRYNVLLEEAFKNQVQTIIQHEKAEIKECSDLQGKTRCEAAYKLHLCYNHLKTLEA

EQRIREILERTEAENEGFGPEGSDFIDGIQHSGEAMTTAKSE
>DmelOBP56d
MKFLIVLSVILAISAAELQLSDEQKAVAHANGALCAQQEGITKDQAIALRNGNFDDSDPKVKCFANC
FLEKIGFLINGEVQPDVVLAKLGPLAGEDAVKAVQAKCDATKGADKCDTAYQLFECYYKNRAHI
>DmelOBP56e
MKVFFVFAALAALSLASAVGLTDSQKAEAKQRAKACVKQEGITKEQAIALRSGNFADSDPKVKCFAN
CFLEQTGLVANGQIKPDVVLAKLGPIAGEANVKEVQAKCDSTKGADKCDTSYLLYKCYYENHAQF
>DmelOBP56f
MKVFLLFIFISAIWLQAFCMKSSEKIKACLKRQLGYTITENTKFDAKEDSLQSKCFYHCLLEVKGVIAN
DAISSEQPRKVLEKKYGITDTDELEKAEEKCHSIKASGKCELGYEILKCYQSITKH
>DmelOBP56g
MRATFALTLLLGCLSGILAQQANIDSSVSKELVTDCLKENGVTPQDLADLQSGKVKAEDAKDNVKCSS
QCILVKSGFMDSTGKLLTDKIKSYYANSNFKDVIEKDLDRCSAVKGANACDTAFKILSCFQAAN
>DmelOBP56h
MKFTLFCIALAAFLSMGQCNPDFRQIMQQCMETNQVTEADLKEFMASGMQSSAKENLKCYTKCLME
KQGHLTNGQFNAQAMLDTLKNVPQIKDKMDEISSGVNACKDIKGTNDCDTAFKVTMCLKEHKAIP
GHH
>DmelOBP56i
MHFFTCCALLLVVVTLPTCFVQAGPIKDQCMAAAGITAQDVANRHETDDPGHSVKCFFRCFLENIGII
ADNQIIPGAFDRVLGHIVTAEAVERMEATCNMIKSETSHDESCEFAWQISECYEGVRLSDVKKGQRTR
NHRG
>DmelOBP57a
MLKLWLICILTVSVVSIQSLSLLEETNYVSDCLASNNISQAEFQELIDRNSSEEDDLENTDRRYKCFIHCL
AEKGNLLDTNGYLDVDKIDQIEPVSDELREILYDCKKIYDEEEDHCEYAFKMVTCLTESFEQSDEVTEA
GKNTNKLNE
>DmelOBP57b
MFIYRLVFIAPLILLLFSLAKARHPFDIFHWNWQDFQECLQVNNITIGEYEKYARHETLDYLLNEKVDL
RYKCNIKCQLERDSTKWLNAQGRMDLDLMNTTDKASKSITKCMEKAPEELCAYSFRLVMCAFKAGH
PVIDSE
>DmelOBP57c
MFNTRLAIFLLLIVVSLSQAKESQPFDFFEGTYDDFIDCLRINNITIEEYEKFDDTDNLDNVLKENVELK
HKCNIKCQLEREPTKWLNARGEVDLKSMKATSETAVSISKCMEKAPQETCAYVYKLVICAFKSGHSVI
KFDSYEQIQEETAGLIAEQQADLFDYDTIDL
>DmelOBP57d
MLDQLTLCLLLNFLCANVLANTSVFNPCVSQNELSEYEAHQVMENWPVPPIDRAYKCFLTCVLLDLG
LIDERGNVQIDKYMKSGVVDWQWVAIELVTCRIEFSDERDLCELSYGIFNCFKDVKLAAEKYVSISNAK
>DmelOBP57e
MSLRLVPHLACIIFILEIQFRIADSNDPCPHNQGIDEDIAESILGDWPANVDLTSVKRSHKCYVTCILQYY
NIVTASGEIFLDKYYDTGVIDELAVAPKINRCRYEFRMETDYCSRIFAIFNCLRQEILTKS
>DmelOBP58a
MKQLIFLLICLSCGTCSIYALKCRSQEGLSEAELKRTVRNCMHRQDEDEDRGRGGQGRQGNGYEYGY
GMDHDQEEQDRNPGNRGGYGNRRQRGLRQSDGRNHTSNDGGQCVAQCFFEEMNMVDGNGMPD
RRKVSYLLTKDLRDRELRNFFTDTVQQCFRYLESNGRGRHHKCSAAREL VKCMSEYAKAQCEDWEEH
GNMLFN

>DmelOBP58b
MLRIGFVICVIISLRLNGLVAVRVHCRHMERIHEENIHHCCKHQDGHDDVTESCAKQTNFRLPSPNEE
AIVDVTVDQAMVGTCWAKCVFDHYNLMENNTLDMDKVRSYYKRYHQTDPEYATEMLNAYEKCHT
QSEEATEKFLSLPIVRAFSTAKFCKPTSSIIMSCVIYNFFHNCPASRWSNTTECVETLAFARKCKDVLTT
>DmelOBP58c
MKCTILLSFFSLIWFAGGIKIDCENTEAINEDHIHYCCKHPDGHNDLIEGCARETNFTLPNQNEEALVD
ITADRAIRGTCFGKCVFSKLNLMKDNNLDMDAVRSLFTERFPDDPEYAKEMINAFDHCHGKSEENTS
MFLSKPLFKQMSKQFCDPKSSVVLACVIRQFFHNCPADRWSKTKECEDTLAFSKKCQDSLATL
>DmelOBP58d
MVNIVCYWTFLILVAVSKAQDNEETTAVAISSGDLTEDKCNTSRAGCCSELYIGEEEDLVKCFVIHSPKL
PVDGDADIGKTLRFLSCFVECLYKQKKYIGKSDTINMKMVKLDAEKTFVDRPKEKDYHIAMFEFCRKD
AVGVYNLLKASPGAKVLLKGACRPYLLMVFMCISDYHQKHECPYFRWEGTAKAGTKDMCENAKAE
CYQIDGITLPTKSPA
>DmelOBP69a
MVARHFSFFLALLILYDLIPSNQGVEINPTIIKQVRKLRMRCLNQTGASVDVIDKSVKNRILPTDPEIKCF
LYCMFDMFGLIDSQNIMHLEALLEVLPEEIYKTINGLVSSCGTQKGKDGCDTAYETVKCYIAVNGKFIW
EEIIVLLG
>DmelOBP76a
MKHWKRRSSAVFAIVLQVLVLLLPDPAVAMTMEQFLTSLDMIRSGCAPKFKLKTEDLDRLRVGDFNFP
PSQDLMCYTKCVSLMAGTVNKKGEFNAPKALAQLPHLVPPEMMEMSRKSVEACRDTHKQFKESCER
VYQTAKCFSENADGQFMWP
>DmelOBP83a
MALNGFGRRVSASVLLIALSLLSGALILPPAAAQRDENYPPPGILKMAKPFHDACVEKTGVTEAAIKEF
SDGEIHEDEKLKCYMNCFFHEIEVVDDNGDVHLEKLFATVPLSMRDKLMEMSKGCVHPEGDTLCHK
AWWFHQCWKKADPKHYFLP
>DmelOBP83b
MVKYPLILLLIGCAAAQEPRRDGEWPPPAILKLGKHFHDICAPKTGVTDEAIKEFSDGQIHEDEALKCY
MNCLFHEFEVVDDNGDVHMEKVLNAIPGEKLRNIMMEASKGCIHPEGDTLCHKAWWFHQCWKKA
DPVHYFLV
>DmelOBP83c
MQMKSGILIALCLCLSLNEGLALLEHEGETINRCIQNYGGLTAENAERLERFKEWSDSYEEIPCFTRCYL
SEMFDFYNNLTGFNKDGIVGVFGRPVYEACRKKLELPFESGESSCKHAYEGFHCITNKEF
>DmelOBP83d
MESHPFTVIDNMPNISPSAKDAMKDCLQDVHQDEWKSFDAFAYYPVNEPIPCFTRCFVDKLHIFEEKT
RLWKLEAMKQNLGIPAKGARIRTCHRHRGRDRCATYYKQFTCYAMAV
>DmelOBP83e
MSSPRAVLVSLFLICSQALADLSGDAQTLEKCLRQLSSPESIAGDLRKLERYSSWTREEVPCLMRCLARE
KGWFDVEENKWRLKQLTEDLGADVYNYCRFELRRMGSDGCSFAYRGLRCLKQAEMHAGTSLSTLLQ
CSRQLNATNVELLQYSKLKSKEPIPCLFQCFADAMGFYDPDGNWRLENWKQAFGPSGNEDQSSGAD
YSGCRLSGTQREVALSKCSWMYHEYKCWERVNGNKLVEDNEEQ
>DmelOBP83f
MQSQSLLLIVAAVATFLVAQVRAQWLPLLMETTAKFLLKDHADAEKAFEECREDYYVPDDIYEKYLNY
EFPAHRRTSCFVKCFLEKLELFSEKKGFDERAMIAQFTSKSSKDLSTVQHGLEKCIDHNEAESDVCTWA
NRVFSCWLPINRHVVRKVFA

>DmelOBP84a
MYSALVRACAVIAFLILSPNCARALQDHAKDNGDIFIINYDSFDGDVDDISTTTSAPREADYVDFDEVN
RNCNASFITSMTNVLQFNNTGDLPDDKDKVTSMCYFHCFFEKSGLMTDYKLNTDLVRKYVWPATGD
SVEACEAEGKDETNACMRGYAIVKCVFTRALTDARNKPTV
>DmelOBP85a
MSPGSVVFSMFLTRPSLDKGNSECRKSLNLPAHRKFNFAELYTINMCIEECNFIGCGYIEIDPPFRLDLA
NIRTNLQTIAPQPQNESIPFLVDAYRKCELFRSSHGRRFTLHLPDIEFIEEPCNPFALQITICVRIHAMQKC
PSEFYVDSDECRLAREYFTQCVGDIETNLA
>DmelOBP93a
MKTSNKIVFLLLQLNIWQLSSCCDVQKNDKAINSCRKSLLGNNSTNSNGEVRNLKSDKVALHACIAE
CSFRTNGFLLSNGTVNTQALQKSYQQRYKNDPNMSQLMLKSLNSCTDYARKRVQEFQWMPKKGDC
DFYPATLLACVMEKVYINCPTSKWKNTSDCTAMWKYLVACDDVASNKKK
>DmelOBP99a
MKVFVAICVLIGLASADYVVKNRHDMLAYRDECVKELAVPVDLVEKYQKWEYPNDAKTQCYIKCVF
TKWGLFDVQSGFNVENIHQQLVGNHADHNEAFHASLAACVDKNEQGSNACEWAYRGATCLLKEN
LAQIQKSLAPKA
>DmelOBP99b
MLKYLIVALALCAVAHADDWTPKTGEEIRKIRVDCLKENPLSNDQISQLKNLIFPNEPDVRQYLTCSAI
KLGIFCDQQGYHADRLAKQFKMDLSEEEALQIAQSCVDDNAQKNPTDVWAFRGHQCMMASKIGD
KVRAFVKAKAEEAKKKAA
>DmelOBP93c
MNHLRLEIICWSCLLIAMAVSTEAASVWKLPTAQMVYEDLEKCRQESQEEDAATLRCLVKKLGLWTD
ESGYNARRIAKIFAGHNQMEELMLVVEHCNRMEQDTSHLDDWAFLAYRCATSGQFGHWVKDFMSQ
KEVER
>DmelOBP93d
MKVLIVLLLGLAFVLADHHHHHHDYVVKTHEDLTNYRTQCVEKVHASEELVEKYKKWQYPDDAVT
HCYLECIFQKFGFYDTEHGFDVHKIHIQLAGPGVEVHESDEVHQKIAHCAETHSKEGDSCSKAYHAG
MCFMNSNLQLVQHSVKV
>AaegOBP1
MNGSVVFVLSALVSLSVGDVTPRRDAEYPPPEFLEAMKPLREICIKKTGVTEEAIIEFSDGKVHEDENLK
CYMNCLFHEAKVVDDTGHVHLEKLHDALPDSMHDIALHMGKRCLYPEGENLCEKAFWLHKCWKE
SDPKHYFLI
>AaegOBP2
MMEQLMLAVLLAVFLGLVADVTMAAQIKDNLELPEYYKRPAKILHNICLAESGAMESKLKQCMDGV
LHDDREVKCYIHCLFDKVDVIDEATGQILLDRLAPLAPDNDVKDVFNHLTKECGHIKLQDSCDTAYE
VAKCYFAAHDQVVKFCHLLMADVTS
>AaegOBP3
MIRFIVFVSSCLVAVSIADVTPRRDAEYPPPELLQALKPLRDICQKKTGVSDEAILEFSDGKVHEDEKLK
CYMNCLFHEAKVVDDTGHVHLEKLHDALPDSMRDIAMHMGKRCLYPEGENLCEKAFWLHKCWKE
SDPKHYFLI
>AaegOBP4
MGLHKVKLLFHVLLAVMLSLHTSESKSTMEQLAKASEMMRGVCVGKTKAPLDLIDGLGRGEFVENK
DLKCYANCVLEMMQAMRKGKVNADSAIKQVDLLIPPEIGEPTKKAFDMCRNSADGIKNNCEAAWA
LVKCLHQKNPKYFFA

>AaegOBP5
MFQKFWILLAFVGPALTHSDDYYDSNRRSRGYNERCYQHEQFPEPSECCTRPLWINQYLVRPCRFSNV
QRDGYRQEHEACSVSCGVYRINMEMLNNQVNSVRIFRPARLRAYGDEDWKRTVAAALKLCKKRITS
MVGSRAREGREAELCEEANDVFADCLDGQLFLQCPARVFIRTEGCELAKSHLLDGCPYRSLTDTTERH
RNDWYDRNQWNGASNGGYDQRNGDQYDDEYEDVNSGHQTRNGGGYNNQNQNGYDRHNNDR
NRNNGQW
>AaegOBP6
MKRLASLLLLFLTVRAEVSTQHCAVAKLPCTLHSECLQYLNSNDDGPENCAYRCIALTARFWDDQKA
DVIRTISRFYLTDANDDDFRNRTEQCLQETQETFPVTESCQRASCAFSCYNDQFGEVIAVRPSFIPFTALE
HRRIVRECVDILQIGPQSRQAILDEGLMEVPEGRCLLRCVLLREGLYNDWRGPRLGSLWVQTEGYEDR
FFDTAQKCYPLLKMQTLEPCELAARFAAECLPSRVPFVETVFAALASNQ
>AaegOBP7
MILHLRILFAVIALVSFCSCNQHKIVQKSLGKANDECVSYGSSEQCLARCVTLVTRDWNETVGLSSVYD
RFYQPDPEDLCNTNRTQRCLEALQSTVAPEDKCLRAAGSVQCYLDQYGQVDMATSRFVKSAPVQQQ
QIIWECGAMLGYSGDQILRSIDDKDYSMQETRCLYRCYLIRSGMYTDEGGLNMERFYVACGGYEDEFY
RNVTECAARVRSSTRCDDRCTLAQRLASECIGTRYDQTLTPGPATIDARDGSSVTYAVFQNYAGRDMT
NTFVLNQR
>AaegOBP8
MKCLVLISLLAVGSQAFFTPEQHEVAKRLTMACATEIGEGLPDNVGNRFREGDLTLTDDKSKCFMKCV
FGKVGFIDDAGTVNKEVLVEKLSKGNTQAKAEMFAEKCNMFEGANGCEKAHGLFECYWKNKEIFA
>AaegOBP9
MLKLVVALLSVTIALNQIKAFTLQQRQQGDIYAIECIAETGVNPASVALLRVGDFSSNDKRSKCFIRCFF
EKEGFMDSKGNLHTEKIADALAGDFNREKVETVLANCLTKEKTACETAFRMYECFYNHREGL
>AaegOBP10
MTSFRLANLTVFLVLLFCFMRGVHSADDLSKIPEIKGYELHCIEASGITESSAKKLRNGDDIASPDQSIKC
YVQCFFSKLRLMNEKGVVQKDKVLSLLGKLMEEDKAKKLAEKCDLRRTNPCDTAYAMYDCYRQNK
AKLL
>AaegOBP11
MRLLISIVSFALVGAALSVPQQANLEDIGKIRNGETYALECLLASGLDVSSLKSLQTGDFSNGDRVKCLV
KCFFEKTGFMDAEGNLNEEAIVTQLSQFMPKDQVETLVKNCKIEGTDACDTAYQATECYFKNKAGLF
>AaegOBP12
MKSFVCVVLVAALIGVNALTEEQLKKADEFASECLEKSNGLSKETVGKLRSGDFANVDQDSKCFVKCF
LERAGFMSTDGNLVADYAIERLSLDREKSKVEALVMKCSMQMDDPCETAFRAFECYYNGKASLL
>AaegOBP13
MKTFAAIVSFALIAGCMAVTEDQKEAARQLAGKCMQQTGTSEESVQRLRNGDTSGADDNTKCFVQC
FFQGAGVVDGEGNMQEAFVTEKLASEYGQAKAEEVVQRCRNNSGANACERSFSLLQCYIANRASLM
>AaegOBP14
MKTIAAIASFALIVGCMAVTEEQKEAARQLAGKCMQQTGTSEESVQRLRNGDTSGADDNTKCFVQC
FFQGAGVVDGEGNMQEAFVTEKLASEYGQAKAEEVVQRCRNNSGANACERSFSLLQCYIANRASLM
*
>AaegOBP15
MKSLVASGVALLMLASSIVPGCAQDFKGAIDECTKEFEMDMDVVVSLKYGDFSERDPLIECFTECLMK
RSGFMFDDFSYNKTLIIGFAGRYLEPEGAQYVYDNCVDKFGTTVCVTGFEMYQCIHETAVAEWVESNF
>AaegOBP16

MIVIVVLVALSSEVLGAGQHDAVFKSIESTGKECARYLNNDGTGDCNTHCVGVIDHVWNDTVAMFTR
NYERFFVPAPEDLCYQNRTQRCLSQVDQVVPVSDKCARARQLGQCYADQYGQLNASQLQYRPMTN
LQYNRVFQQCSSMLGLSNDVLKDIATKGVDSVPAFACLVRCTMIRMGLYSDNEGFDLTLATGQCGKY
NPALDPVPCQAKVKAEECDRCKRTVRIANECLGLRLNVKQLEENQPPQLVFNIEVYDFNCIILCDFEIN
F

>AaegOBP17

MKSIASAVLLLIFSVLVHSQSIKDLVEECKQTVPISEELEKSFLKLEFPPEEKTTHCLLDCIGKSLKVMDEK
SGINLAVVTKLLQEVEPEGVIGEEQVRCATEAATSKEDQCTMAFKLYQCFEKEFLALMKMKLDQGE

>AaegOBP18

MTTHRLFIAATLLVLLVSLAYASEVLTKRQQYDQHKLMCGKIVRSTKEDRELYSQSQYPETHDTACFLR
CVSILSGSYDDETGVNLDVLYDVYGKGTTAEEYAEESKACLALRDEVECYCMKAYKPLMCLREQFKKR
NTA

>AaegOBP19

MLKAMTSNRCWIAVAIVCLMGVAAQAGPDFRTKREQYDHSKQMCGKILRTPAADLEHYLRSDYPES
HDTACFIRCVSILNGGYDDETGVNMGVLFETYGGSLTKEEYADEAKECLALRDEVECYCMKAYKPILC
LKEQFKKRNVL

>AaegOBP20

MKPGVKLSLLLLIGLMALLDSTSGCSMTNNDGVEQREALLADPSTAPARSMKDYSVEDIYADCNKTF
AISMDFLNELNDTGSFPDETDKTPMCFIRCFLQKGEIVTSDDKINKEQAVALGWVKNGETIDDCLQEL
TGNPCERAYFLMRCVSTRHLVEGRSKDSKKR

>AaegOBP21

MQIECIIVLTSLIAAASAGWRLQTVDDLLRNRNKCVKILNMQDDLQEEFGLFDFPDQDSAKCVFKCIM
NRMGLFSDKRGPHVGRLVKQMKFASMSSTKAIRDEILNCAYQDMEMDPEDVCDRAYALYQCIQNSN
LLQLKSPETVKT

>AaegOBP22

MKVFIAVFALIAVAAAEFTVSTTEDLQRYRTECVSSLNIPADYVEKFKKWEFPEDDTTMCYIKCVFNKM
QLFDDTEGPLVDNLVHQLAHGRDAEEVRTEVLKCVDKNTDNNACHWAFRGFKCFQKNNLSLIKASI
KKD

>AaegOBP23

MLKLVLCLSALGLVACYDFKDSFYNELVLEEILDSEDAPSLMDRFKRSNPEMMDDKCKRNHRHKCCN
DANGENMDKFRETKKQCFNEVRSKDRSARGMMNPVDMFDCEKMNKTKQEYICAVECVGRKFDIID
KDGNLLTTDKLVKFTKDNFAADPWQETVVDGLVESCLKEVAEKNEKMKSSGEHTTCNPSSSNFGYC
MWRQMTLACPKDKQDTSKKCERMREKFANNESFSMYHKHDFDDK

>AaegOBP24

MNKLILVAIVATVAIGTSQAFVPVFHRFRRSPSVRCCNDGFEDSINHEKVVAVRRTCAEELGLNEMSEE
ELLKNRENLVCLVECIAKKHELADETGDLLHEDLAKAVKEHFSVAEWKAPLLDDFIKQCFDHAEEEH
EKHPTEGGKCNPEGFDFSYCLWRHFTLACPEELQDDSERCEAIRRKLKSDEDVGFWNNDFDETK

>AaegOBP25

MNKLTLVAIVATVAIGTSQAFVPVFHRFRRSPSVRCCNDGFEDSINHEKVIAIRRTCAEELGLNEMSEEE
LLKNRENLVCLVECIAKKHELADETGDLLHEDLAKAVKEHFSVAEWKAPLLDDFIKQCFDHAEEEHE
KHPTEEGKCNPEGFDFSYCLWRHFTLACPEELQDDSERCEAIRGKLKSDEDVGFWNNDLDETK

>AaegOBP26

MADDMAYQHFKMCDKATKKSEPPVSSENQSHLSLPIRVDAKRLQRRDDDDADQLFANQLTGSAASQ
QVQVKLGTYKNDKQNSLIIHSGGNCRTSITPTTREIGESEKLCRMLKLVLCLSALGLVACYDFKDSFYN

ELVLEEILDSEDAPSLMDRFKRSNPEMMDDKCKRNHRHKCCNDANGENMDKFRETKKQCFNEVRS
KDRSARGMMNPVDMFDCEKMNKTKQEYICAVECVGRKFDIIDKDGNLLTTDKLVKFTKDNFAADP
WQETVVDGLVESCLKEVAEKNEKMKSSGEHTTCNPSSSNFGYCMWRQMTLACPKDKQDTSKKCER
MREKFANNESFSMYHKHDFDDK
>AaegOBP27
MKTLSVIILGAWLVHLGGVMSSMTFEDMQETAKMMRGICQPKYGIPDDVAENASSGVFPDSREFKCY
ASCLMDLTHTAKRGKLNYEAAVKQITMLPDDFREPFRVGLDSCRNAADGIDDYCEVAYTLLKCFFKA
SPKFFFP
>AaegOBP28
MKVFAGLLIAAIAASASAVYYPPLAPSDVEESHFAYQLKSFRQELDECAEYLQVSPGSVENLVAYNYVT
DDPSLKCLIRCAGINAGWWSVGGNNSGLQPPVIESYFAPGCDDTCYVKRTQDCVSANVVPCQDDCS
QAYQAFLCYYHQYGNLKSSEEYIPLPQLDAVQAAIDCMLILRTPKELLEQYVQGVFPDVPETQCLYRC
QYLAEGLYDGVTFNLTRNYIREYAVPSPQIKDPATQACVDSALSSSSCNECARFWAGRGCLKNYGVPN
HSGSYFQVAAGLVLNQRTCLDEDLNPHIFTCCSDVHGSCKAACESNLSKKL
>AaegOBP29
QQDAVQAAIDCILTLRIPKELLEQYAKGVVPHLKSFRQELDECAEYLQVSPGSVENLVAYNYVTDDPSL
KCLIRCAGINAGWWSVGGNNSGLQPPVIESYFAPGCDDTCYVKRTQDCVSANVVPCQDDCSQAYQA
FLCYYHQYGNLKSSEEYIPLPQLDAVQAAIDCMLILRTPKELLEQYVQGVFPDVPETQCLYRCQYLAEG
LYDGVTFNLTRNYIREYAVPSPQIKDPATQACVDSALSSSSCNECARFWAGRGCLKNYGVPNHSGSYF
QVAAGLVLNQRTCL
>AaegOBP31
MGVLIGLFVTAIAATASAVYYPPMAPLDVEESHFAYQLKSFRQQLDECAEYLQISPGSVENLVAYNYVT
DDPSLKCLIRCAGINAGWWSVGGNSSGLQPPVIESYFAPACDDTCYVKRTQDCVSANVAPCQDDCSQ
AYQAFLCYYHQYGNLKSSEEYIPLPQLDAVQAAIDCMLILRIPKELLEQYAQGIVIKGPETLCLYRCQYL
AEGLYDGAAFNLTRVYIREYPVPAPQIKDPATQACVDAALAAPHCNECVRFEAGHACFDAYGVPNH
TTPIFQVAAGLVLAQRTCLDEDLNPRYNAGGSAPQPTPAPTPAPTAAPTPAGCVYNCGA
>AaegOBP32
MSVLICLFITAIAATASAVYYPPLTPSNVEESNFAYQLKSFRQQLDECAEYLQISAGSVENLVAYNYVTDD
PSLKCLIRCAGINGGWWSVGGNNSGLQAPVIESYFAPGCDDTCYVKRTQDCISANVVPCQDDCSKAY
QTFLCYYHQYGNLKSSEEYIPLPPLDAVQAAVDCMLILRIPKELLEQYAQGVFPEVPETQCLYRCQYLA
EGIYDGVTFNLTRDYIREYTVPSPQIKDPATQACVDNALASSSCNECARFWAGLACFRDYGVPNRSVG
SFQVAAGLVLGQRTCLDEDLNPRYNAEGPAPPAPTSAPTSASTPAPTPAPTPAGCMYNCGS
>AaegOBP33
MSVLTCLFITAIAASASAVYYPPLAPSDVEESNFAYQLKSFRQQLDECAEYLQVSPGSVENLVAYNYVTD
DPNLKCLIRCAGINGGWWSVGGNNSGLQPPVIESYFAPACDDTCYAKRTQDCLSANVAPCQDDCTQ
AYQSFLCYYHQYGNLKSSEEYIPLPQLDAVQAAIDCMLILRTPKELLEQYVQGVFPDVPETQCLFRCQY
LAEGLYDGVTFNLTRDYIREYAVPSPQIKDPATQACVDSALSSSSCNECARFWAGVDCLKNYGVPNLST
SYFHVAAGLVLNQRTCLDEDLNPPPAPAPTPGCMYNCGS
>AaegOBP34
MIKIRIVTLLVAVLLLETLRPSDAAMTMKQIKESMETMRKACAPKFDVPETTLNDLKAGNFRPDASKD
EKCYAKCIAQMAGTLTKKGEISFSKTTAQIEALLPTELKAPAKEALKACKEVHTDYKDSCDKVYYSVK
CAADFNRDVFIFP
>AaegOBP35
MKFFVAIAVVALAAGAWALTIDQQKKAEAYAAECVKSTGVAPDTPVKLKKGEFAGADDKTKCFSKCV

LEKAGFMNEKGEIQEKTVIDKLSVDHDKAKVEATLKKCNQKGANACDTAFKMTECFYNTKAGLV
>AaegOBP36
MVRPCLYYCCILIAIFCWVQSWLVGAAPQKAGEFSRSMGIEMTASQHGECVTETGVSEESIARFNGPEI
FEDDDKLKCYMDCMFRKFGATKPDGEVDMIEVYHKIPKDFNSVALIVNNKCRDAIQGANQCERAFS
HHKCWKQMAPEVNHVHS
>AaegOBP37
MYRKTLLAFFFFLFLSCGDAVQNLTALRGSDYPPMYLINLVKSALERCHQLIDIEDSVIVRFRDDGDYE
GTEQLGCYLHCVFREKGYWIPEKSEVDIMKILDIVPKDFEQPALKMGLRCLKVKGDDDCSRSLWYHSC
WKKNDPAKVES
>AaegOBP38
MVTTLLLLFLVGVINCQEPRRDANYPPPELLEKMKPMHDACVAETGASEDAIKRFSDQEIHEDDNLK
CYMNCLFHKAGVVNDNGEFHYVKIQDFLPESMHLITLNWFKRCLYPQGDNLCEKAFWLNKCWKER
DPVHYFLP
>AaegOBP39
MYVVNLVLVLLSLEILSTSDAAMTMKQLKNSLEMMRKACAPKFNVVEASLDELKAGRFANEADKEL
KCYTMCIAQMAGTLTKKGELSLSKTTAQIEAMLPQEIKAAAKEALNACKDIQSGFKDPCEKVYFSAKC
AAEYNPDVFFFP
>AaegOBP40
MTRLVSSIICVWASLLLSISAQYLQNEALLQAQATCVEYLGIPEARLEQYNISVYPPDRDTMCMIRCAGI
VLGFWEDEQGLLIDGAKQLFPDSGDVDLVAQKVLHCAERKLLSCDPADACARAYYSFRCAMRKFEPS
NSALSTDQKLTPEKFLKAQIVCANILRIPHDHLKLYNQGVYPDDAETRCLLRCIGVRLELYSDAMGPN
LDRLHSEFAIDQPLEEFKTRATLCCEANRPLIQDYCTAAYRNLYLCFREHFNAFTSQNRQTLLSHTSSPQ
TCIDLESDILLYGDDV
>AaegOBP41
MKHLVSFVLLALAIYPVHSARRYRIAAEECVQYLKICPTRLEQYLKFIFPEDRETMCFMRCVATKLNLW
CDRKGLNWAVLEDRICPSVREKVEACVCRKLDLIDPYDHCPRAYYAFRCLRNYLQEIFLFKNLDRDFD
DGYIVKVPSSKELIVPSCASCSNSFNPLTITEMTQKLLQCAKKCQLCSLNLCDRTTDPVVETPEFQCTVY
CASICTGVYSEQKGILMDNLYAQLARCETRESFDYRLGLCFGRNALPEGSSPQAVVFQQYFKCLRGDY
ERFYSSNLEELLQIPGISKYCF
>AaegOBP42
MECCNTPMLLDKDIMMDCYQKYGDQTKKQMKLEGVPRGCCIAECGLNATGLYSNGMIKRDDMTK
MFMDSVKDMPEWQMLVRDTLDECFKMAESKMDEIQAGAMLEPSFEGEKICHPISGTILRCMGMNLF
VKCPAGVYNESDECNQLKEYSKMCPIM
>AaegOBP43
MKIVIATCFLVGLLLRFLVAGDDDVCKNGSPTNKSLWDCCNMPNLVNQDIRADCHQKYGEQTMKQ
MKLEGTPRGCCIAECQLNATGLYADGMIKRDEMTTMFMDSVKDTPEWQPMVRDLLDECFRQAEAN
KDIIAAGAMLEPSFEGEKICHPISGAIMRCMNKNLFLMCPKESFTEGPECTQLMDYFKMCNGI
>AaegOBP44
MESKTFHFLLPLLCTLASYTEALDHAAILKSPNELQLECSKYLPSIDVSRNVDCTDRCIGLVGRFWNDSI
GRPAQTIARYYQPDTGSQDYITRTDQCLCEKVLTVPRNAYCQRASSGLQCYRDNYGQLLTGTPQFVPV
TEIRAAQIFWDCAQMLQISRDRLTQIFKDGYNKTSEGRCLIRCFLVRAGLYSDCQGPNIGRFAVQCEGY
SAEYEQAAVMTG
>AaegOBP45
MELKTCCIILPLLCTLTSYAEALDHAAILKSPNELQLECSKYLPSIDVSQNVDCYDRCIGLVGRFWNDSI

GRPAQTIARYYQPDTGSQDHITRTDQCLCEKVLTVPRNAYCQRASSGLQCYLDNYGQLLTGTPQFVPV
TEIRAAQIFWDCAQMLQISRDRLTQIFKDGYNKTSEGRCLIRCFLVRAGLYSDRQGPNIGRFAVQCEGY
TAEYEQAVVSCYDRLKKESLDSCSLATRTMDECIQGNQFSSSDIDGLEKLEVQ
>AaegOBP46
MSGSISLIVLAVVALAGQVLGRHDATFKSFGSTSGECSRYLNNDGNGECNIHCVGVIGHAWNETLAKF
TQNYAGYFVPDPQDDCYQNRTERCLLQVDNAIPVYDKCTRASKLGQCYADQYGQLNAIQPQYVPMT
DLQYTRVFLQCAAILGLSNNDLNAMVQQGAYNTPAGACLLRCTLIRMGLYTDDAGIDVALATRQCG
LYNATSDIAQCQAKVQAEECDKCKRTTRIAKECLNMHYNVRNVGDSYGLELYGVDTCYSSCSFFYCY
YYACPYLSYYNTNYAGSSSYSGSSNSLTFAG
>AaegOBP47
MHLSALFFTVVSFLGSFCFAINPCIEGPPVNKSPSECCTTPALIDPPLMMKCFQKWGEQTKRQSKMDGI
PRGCCVADCAMEGTKLISKGKFNREKARKVFMAVVKDQPQWQPIVNETLDACFKQADENMAEIEA
GAKLKPSYKGEKICHPISGSILRCMNMKLFSKCPNDLFNSGPECDQLKLYHEKCPLN
>AaegOBP48
MKATVTSVLVLLAISHATLADPAAPDNVPASCLNKNFNVDPFECCKTPKLLDEGTVKECVHSFPPPQN
AQDEIKPDCMSECVMNSTRIFDRRQNVNDAKAMETFLEKLNGKSVWAEIVQKAVKQCLDDADNRK
EEFSRDMKALQQKFPKERICSPAAGFIMECVHVSVYKNCPASIFKDNLAGCPAIKKHLNVDNCPFYTIF
PEKKAPKPVKRH
>AaegOBP49
MIYSQPPPDDKACFQGYKVDANGCCELPRFVAREINAKCDEEFKPLSPRLPPEVQAYEGSCVIECLFNV
TGMFKDGKLQQDKIAQQLKKTIGADRNFAPLLGGVVTDCYRLVMDNPANSFKPIPVKPGRPGCSFIP
QAYMNCVKSELFENCPKANWTAADGCDLLKQKLNVGCSYYSIMIGKKGLKS
>AaegOBP50
LLIRFNTNLKTQVVAQLPQEDISCYMGNVKIARECCLMPRFIDKTVDDACTAEHKNPGPRVPPWTAK
TEGSCVVECVLTRIKSFSNNIIDKEATKLSFGKSIGIKTFFGAVTNRSVDLCHKRILNNTALQLANPVSH
DSNRTACSFVPTVFLDCFKENVFMNCPKQKWINVPECNALRSKISSGCTFNAIKGYSNSTHF
>AaegOBP51
MLVHLLPTLIVTLLGIGTVVAQPRPDDPSCMEGNQRKAHDCCRMPMLVEQSVMNRCMTENPMTPPV
PGVQRTEGCCIAHCVLTTLNAFRDNLIDAAAAKRALTQSMGANSSFVSLVSGVVDECVNLVHGNAA
YKVAPVASTPGRPGCSFMPEGFVNCIKGRFFQQCPTAEWTRDAACDQLKQKLTAGCSFGSLMG
>AaegOBP52
MFKLGFLLILVSCLAISVQCVGFDPSCFQSSSSKKADDCCLLPKFYDSQMVSDCLTSISKSTNDVEKYQC
LVECIAKKLNLFKGNTLDREATMQLYKARIGSVPHFAPIMDNIFQQCYDGMAVYAAQDRSDPTKCSA
LPMMLLNCIQTRLFQNCPAKLWQGGPECQELKEKLLEGCPYAAIASF
>AaegOBP53
MLNLWLMVLSFAVTTHNSTWDKSCFELKTSKRADDCCDIPGSFDEALLKRCYDEQKASKNEQEAIKCI
AECVARELGAYKNHTLIRENSRLVFESTIGSDPNFRPVLGDVFEKCFNRITAIEAQETYKNATCHFAPAF
MLNCVESGLFENCPVSIWNEGVGCDELKEKLEQGCPFFAISETL
>AaegOBP54
MFIDAYSILKLSCNDGPVDKSCFELRTTKRADDCCKIPDILVESDESMVRRCFAQQNKTLDEHETAKCA
AECIARELGTFKNGALDKELAKKVLLGRLDKDKNFKPIVGGVLDKCLGRINAVIEKESKRNGTCNATA
NFLFDCAEQGLFENCPSSVWDSNDGCVELKNKLAQGCPYSAIAE
>AaegOBP55
MTKRMELVLFGLFAVVTLFQTGLGGVGVEGKATVEQMTKTGEMIRNVCIGKLKVAEDLVNMLGDKQ

FPDNKELKCYVNCIFEMMQVVKKGKLNYDAAMKQIDTIMPDELAEPMRIALNACRTASDGIKNNCD
ASYAIAQCVAKNNPKFVFP
>AaegOBP56
MEKTGKLFRQVCQPKHKLSDDILEAGKNGVFPDTKNFKCYISCLLDMMQVTKRGKISYEKSLKQIDQ
LLPDDMKPDFRKGLEACKDVASGIKDQCDSAFVLLNCFYENNPQFILP
>AaegOBP57
KFSSALSFCSLQLAWRFVTELQCANSDEEKKAQAKEMMRGMAEECKKKEGATDEDVEALLEDKTPE
TEVQKCFLSCFQHQFQISDGKRFNKDGFMQLSAMMFGEDQEKMATAEEIAEECSSVENADRCQLSVD
IKECVEKAMDKRGIKMEK
>AaegOBP58
SSPFYHALQVCARDLSVPPDRFEQYRLLIFPDEPDTHCFVRCLLLGIRAWHDQTGVRHSALQQYFSPD
DNPVDAYARVQTCLDYVSNSCAATESCTKAYWSLNCYKQQFGSYFFSREQFVPATDIQLAQAMFDCA
DKLDISRSIVAAYRNGNRSELISSRNPCYVRCVAISIDLYDDDAGLQWDHLYVQLGLNEHRENYLEQV
HKVVSELQLSTMDRCAAAAQVIEPFLLTALQQSRTAYRTGIVVETTASITTTEVSVTKPMTTKTPSTTIQ
MTKASTSAPPTT
>AaegOBP59
MKPGVKLSLLLLIGLMALLDSTSGCSMTNNDGVEQREALLADPSTAPARSMKDYSVEDIYAECNKTFA
ISMDFLNELNDTGSFPDETDKTPMCFIRCFLQKGEIVTSDDKINKEQAVALGWVKSGETIDDCLQELTG
NPCERAYFLMRCVSTRHLVEGRSKDSKKR
>AaegOBP60
MILLNMAVVLLEVMLTLAADKPIPRRDAEYPPPFVLEISKKPHKMCVASTGVSEAAIKRFSDEDIFEDD
EKLKCYMQCLFEKLRYTDDKGELHLGKVMDSVPEEYEDIALKMGSKCLKPKGKTQCERAFWYHKC
WKTSDPVVSICDYVFL
>AaegOBP61
MKTIAAIVSFALIVGCMAVTEEQKEAARQLAGKCMQQTGTSEESVQRLRNGDTSGADDNTKCFVQCF
FQGAGVVDGEGNMQEAFVTEKLASEYGQAKAEEVVQRCRNNSGANACERSFSLLQCYIANRASLM
>AaegOBP62
MKIVIATCFLVGLLLRFLVAGEDDVCKNGSPTNKSLWDCCNMPNLVNQDIRADCHQKYGEQTMKQ
MKLEGTPRGCCIAECQLNATGLYADGMIKRDEMTTMFMDSVKDTPEWQPMVRDLLDECFREAEAN
KDIIAAGAMLEPSFEGEKICHPISGAIMRCMNKNLFLMCPKESFTEGPECTQLMDYFKMCNGI
>AaegOBP63
MECCNTPMLLDKDIMMDCYQKYGDQTKKQMKLEGVPRGCCIAECGLNATGLYSNGMIKRDDMTK
MFMDSVKDMPEWQMLVRDTLDECFKMAESKMDEIQAGAMLEPSFEGEKICHPISGTILRCMGMNLF
VKCPAGVYNESDECNQLKEYSKMCPIM
>AaegOBP65
MDTFNAIRNGDFSIRTPFIECFGDCLVKKAGFMNDDLSFNKDVIVKFASRFIKPEDAETVYSQCTADVA
PVLCATAYDVYQCIYENALAKWGTRRNGK
>AaegOBP67
MNESLCRNNTVLARNCCRLPGIINQSIVDDCDDKFPHHAPVKRVEGSCVVDCMYKTIGAFQNGTLD
LDITLQHISQTVGRYPNFEPLVNETVSWCYRNVTENPALQKSVGCSFIPQEMNDCVKKMLFMSCPPSN
WTTKVECDDLKGKIAEGCSYSSLY
>AaegOBP68
MDIFIIGLMLASSVLGQPPAENKTCYQGNQKTAAECCPLPRMMEKSIADMCNSKYKALSPRVPPGVQ
KTEGSCVTQCIFTTIGGYNEKNNTLNIEAIRKAILTTTANAKAFLPLFNSSIDHCYPIISKDPQFLANPVS

PIPEREGCSFLPPALMNCIKIDLFQGDVVSLDLSKAFDRAWRFPILKSFED
>AaegOBP69
MKMSYSHELLFVAMLSAVLHLSSAMDCKEVWERKHETADCCSAPAILNLDNLKSNIEGQEGNKHEK
FFCGVHNLMKEQNLVDDEGNLDVDAMKQNTEGFDDEWKQIAQQAIDHCVQKTESMMADMEQRG
GPKGQCQPTAGMFLMCLGKASIKNCPADKWNSSELCEKVKSGECDKRGHKH
>AaegOBP70
MIRVLLFLTFFVGATLSYDFKDPYFNDFLLEDLMVLQGRPALKKASDSEESQLQYTCCDYLNEENFSKL
QQTQIVCYVENSLLSATKTKSGRAVSPVDMFSCDRLDKLKQQYICASDCVARKENITDDSGNLLGSEV
LVPFVSQYYAPEVFQDEQIKEFVDTCLGESKTDETVANKCNPSSARFGYCMWRKTILSCPNERQDTSPA
CDNLRDKLLYQEAKYLSDETR
>AaegOBP71
MVRVLLFLTFVVGATLSYEFKDPYFNDLLLEDLMVLQGRPALTKASDSEESQLQYTCCDYLNEENFSKL
QQTQIACYVENALLSATQMKSGRAVSPVDMFSCDRLDKLKQQYICASDCVARKENITDDSGNLLGSE
VLIPFVSQYYAPEVFQDEQIKEFVQTCLDESKTDESVANKCNPSSARFGYCMWRKTILSCPTERQDTSPA
CDNLRDKLLYQEAKYLSDETR
>AaegOBP72
MNIIVFLAFLVLAVDTDKSPVDAECIDVEKNADEIRQCCDIPSPLEMENIQTCKEKYQEELGSDVPNLV
ACIFDCHARELGVLKDDLEIDEAKMMEYVSQTPDEDVKKLMVESAKECLKAKGEIMEKAKEHAMKC
HPLAFMMTECIMHAVYSECDKLPNHWKDSEICSKVKNGAEPCE
>AaegOBP73
MQVLNFLCLVLLCLVLEKVAVAEECIKFEDHKDEILNCCKYQPPYPKDDVKECVQEAQGKSGGDKHE
FFACLLECYLPKIGIINGDSIDEDKISEHLQSLDENARDILLAAYKECDESTTGTTRAQCSSYALDLETCV
LQKLDQQCPDEFYNPSEICDKLKSGVEICH
>AaegOBP74
MKLNLALAALIGMVAMVHGQQQINQECFNRPNDKNPMECCRAPNIMPPREELITCMQKFPKPSGPP
TPGSPPPGHNCMAECMLEQQGIMSGGALSKDTATSKLVALVGSSSEWQAVARKSIDTCYSQVSSLGGQ
KDSLGCSVIAGSFMECMPSMMFTNCPSSAWTASAECDQLKAHLQKGCPLMTLFKGPHPH
>AaegOBP75
MKLNFALLTVIGLFAMACSQQPISQECFTRPNEGNPKDCCKAPNVIPPKDQFAECMQKYPKPSEPPTP
GSMPPNHNCLAQCMFEQQGIMADGAVSKDAAISKTVAVMGGSSEWEATTKNVVEACFQKVSALGA
QKDSQGCSVMAGSFMDCMPSMMFTNCPSSAWTASTECEQMKAHLQKGCPIFTLWKGPPPH
>AaegOBP76
MEIFHVGPMHQNGCPGPISQCASQNSNVAKQIDDYRKQCVELSDVSVDSAIKVHSGQVIENPDWSTK
RYVQCFFQKMQFMDENGVMLKDAVVEFFSRIQDESRAKAMVENCDIQKENPLDTAYAVLVCYQGNK
N
>AaegOBP77
MKHSGAIACCLLIAIVAVNAWPSYKRAEVRAHVRNCVKKTGIPGKNALKVLKGNFNDDSSEVKKFM
KCMFQEVGFINEKDELLDNLLIAKIKENLEEDEADELIEKCSIVGDDINDTAFQIYKCYYENHDLPPDM
LVR
>AaegOBP78
MKILEVVVFLTVVALCKADYSDKQKQKLDEFTSKCIEDLDLPKDSDLGKKFKYGQLKEKDDATKKFIS
CSMQKLSFMNETGSILEESIIEFLADKYDRTMAMNVITKCSKLKNESMEDKAAEFYDCFFMQKSFDI
>AaegOBP79
MKLSINLFVILMLTTLFVSTHQLGFKPFSAEKLRDIELICMKLLRQPIAFWYKYLNLEYPDDPITHCHLR

CIGISTGLYGDEFGAHLDNIYEQFKENTLLNRTAWMEEKNNCLAKQFADGLPDDLCKRTFLTFKCFEV
DYLLALSKSDCSKISI
>AaegOBP80
MKWSLKLLVLLTTLFIPSQQIIFAPHSAEEIRILEQGCVKLLLQPSVFWYKYLNLEYPDDPITHCHIRCLLI
AAEFYDDELGAKLDNLFEQHQHDTPLDRTEWTEAKSICLARQFANGVPEDLCKRAYMTFKCFEIEFLI
SITRMDCNKISL
>AaegOBP81
MKAVRFLILLVIIGVFHTIPADAGQLLNKLITVCTQGQNPPADLVQRYRNGEFPNDRNTHCMMRCIAL
NLGVYDDLNGIHMHDTWQMFRRGRPASHEKAFAEQHRQCITQQTKDVPLDDYCGRVYAVYQCYKD
EYEALLRNVRQGAAKARN
>AaegOBP82
MPPLLDENLLHGCKQLHGGEHLTRGLIHERGSCFIECAMNSTGTLVNGVLDQPKIVQLITTRTAGVSS
DLTQVMVASCVKCFLTPLVMGNHSGHPLDSKHCRPAASIFVSCVNMEMFKMCLPEFWTNSDSCNNL
RLHITNCPIPA
>AaegOBP83
MGNHWPSSHSILITVSIVFLFLLLEETWALKCRTEDGPSSDEIRKVIRVCMKRITSESENKSNNEYENYDS
SYSDSNSDEDRESSTEGNTRRQTNGGNQSTNTRGRNGEDMSRGRDSSRSSDDRSRGDNRNRQDNGR
RRDRERDYDYGPMGRRMDDGRNQQGRYKRQYYNDGAQGGYGYNYQQNDRYNRDRNQFMHPNG
NTSSNGTNNTERDRACMMQCFFQEMKMTNNEGFPDKHKVLHVVTKDLRDYELRDFYTDSIQECFH
MISMDNKLKDKCDYSMKFVTCLADRGQANCNDWENEAIMF
>AaegOBP84
MRRFKLASFILTLFATNVICSRHKIVQKSLAGTGVECQQYDPPWNCAVRCQTLLTRDWVDSTGMQSPY
DRFFQPDPNDQCYMNRTQRCLLDKLSTVPRNKLCLRADSSVQCFLNQSGQVIMDQPKFVAPSRLLEN
QIFLECGTMLGFSRQRVWEVLYKGEFTLPEISCLVRCFLIRSGLYDDKSGLNLERFYVACGGYDDAFYH
NVTKCIANVEAAGLCDKCTRAQRLALECVGSQYPIFVPVSQTDIDSTNNAGRDVNNYYTSNFNFNFG
DVISQIGTMVPATGGG
>AaegOBP85
MSCLNLATLILAFFATSVICTRHKIVQKSLAGTDIECRQYDPPWNCAVRCQTLLMRDWVDSTGMQSPY
DRFFQPDSNDRCNTNRTQRCLLDKLSTLPRNKLCLRADISVQCFLNQSGQVIMDQPKFVAPSRLLENQ
IFLECGTMLGFSRQRVWDVLYKGDFTHPEISCLVRCFLIRSGLYDDKSGLHLERFYVACGGYDDAFYHN
VTKCIANVEAAGLCDKCTRAQRLALECVGSQYPIFVPIPLTGKLKLHG
>AaegOBP86
MTIFNALLAILACFSLPTDALQHNAVYKSINSAGPECRTILTRQSPLDCRLRCLSINTGDWDDCSGVPR
TYDRFYVQDPTDVGYQQRTQQCIANVSVSILRGDICAFSARSTECYDANYYDIVLDQLVFVPSKSLQYQ
QTIRDCAGMLGFTEHVISDVLRDDCFALQETRCLLRCLLVREGLYGDQCGAQIDRLYVVTGGFDQLFR
RDVKKCTGRLRAMGLDKCTEAYRVASECFPEDKAILPIFLKNKAILQEI
>AaegOBP87
MMSSGAFILSVVLSVSISVLQTSSLQHSATLKSFNEILSECSRYLPSNDEPCYDRCLGLVGRFWNDTIARP
SVSVGRFYRPDPCDQNYVNRTQQCICDSVLPLPRKDVCLRASRGLQCYRNQYGRLIADEPLFVSVTPL
QSSQIFQDCAQMLQIPRAKLEEIVQQGYSKSPEGSCLVRCYLVRAGLYSDSQGPDIARFAVQCEGYEDA
YEASVARCYQKLKSEQLDKCTLAARTYDECIQANEYSNSNLEILGVLLGIITGLIPA
>AaegOBP88
MQVFAAIVKLVAITLGAIIASISCIEEHSASLKSILSSTAECNLYLPTEALRQECGTRCVSLVNRIWNDTNG
RLSDTIGRFYVEGPQDPCARNRTLQCLEQVTASIPIRNSCKLADASVNCYRNNYGQLDVKSPRFVAFSD

VQQVRILTECAAMLGVSDKLVQVVRNGLQSIPEGACLLRCLLIRQGLYSDQRGPDLKRVSVQCGGYEG
YEQEWRANVTRCVAAVHAERICDKCLQAERIAVDCLQMHLHLYEVRSPKLRQHIPFGVEFYTRANAA
AGSAAAAQARVITYITVEYYYWY
>AaegOBP89
MINQLLITLTTVNILTTSAVEDWRSPQLKSFSSAQQDCAVYLLLSNETVQQYVKSGYPDEFSSRKLINCIL
VQIHAFDELTGIKDHVLTNFFDQPGSCSEYVGRTQECLRTSVPKHCEGQPFEHAYRSFQCYYRNYGSLL
MDTVRFIPYEQVDRIKHLTESFSIVNTSCKALRELSVGQGFIVENIADPMYTLAVRSGFYDREHGLYLDR
LYTQFGKPALLSDATRQCLVRVSQQYQTEPLRLTQLVLQCVESEISTQSLFTETARQVLASNSSYCNVCE
QLPSCVTMTPGVTSAAVTTTRAPLSTSTRPPYPSI
>AaegOBP90
MNAWDDETGIKDYVIRNYFKPADTDPSYKSRTQCCLRDKVANLDRCALFERAYHSFMCYYQNYGNI
VPEAQFIPWYQVDREKHLREVFLIEGITRVQLEEFQRSDALKAKEYPILYYIDFVRTAFYDPSTGHNLER
LYTQFGNPGLLADETRRCLDAVSLQYCDEPVRAYQGFDQCLRNYMTTEELFKTVVAQVLASNIVCR
>AaegOBP91
METRSIFIAVILSITSYVKAEDYEAPRLKTLATIEQECAGYLLLSNETLRSYIAASFPKDSTVQKLVHCFLV
NMNAWDDETGIKDYVIRNYFKPSDTDSSYESRTQCCLRDKVSNLDRCAVFERAYHSFICYYQNYGNLV
PEAQFIPWYQVDREKNLREVFLIEGITRAQLSKFQKSEERNPKEYPILYYMDVIRNAFYDPSTGHDLGRL
YTQYGIQELLADETRQCLDTVSRNFFEEPTRAYQGYDQCLRKYLTCMWKNCCRLLLRRFWNQI
>AaegOBP92
MRCCLILLVSLVALHSSLALNHLEKLRWKTFREAELESAEYLFITHETLERYRSSGYPDEPSVRKLIGAIM
VVLNAADEKLNLIKDYVLSQYFLPNTVDCQYKQHTKECLDRNVATLDPSDRLGRAYQTFQCYYKNFG
GIKEDVGWVPYHYSEVVQMLEDCLYITNASNESLLQYCQGGYATSADYSNVAYCYAVRAGLLDKTTG
FNVEKMYIQLGDDNLNDGDAKKCIAGVVNQYCKEPYRTMRIVVDCVLIYLPGVAGIVIAASNILGNP
PECVIPPSPPPITQPCYNGRCL
>AaegOBP93
MSYQLLTPLVTFIMASSLAAEDWKSPELKSFSSAQQECAVYLLLSNQTVQRYVKNGYPDEFSCRNDSVS
RTQECLRTTVPKHCEGQPFERAYRSFQCYYRNYGNLLKDTVRFIPYEQVDRVQHLKESFSIANTSCAAL
KDFCEGHGFNVAELAEALYVLGVRTGFYDPQHGPYIDRLYTQFGSPNLLSEATRQCVNRVSQQYSTEP
VLITQLFLQCVEDDISTEALFTETAKEILASNQSFCNVCETLTSSVSSSTTEMVTTTTATISTSGAPLLTTTK
GPYPYRSM
>AaegOBP94
MNHQLFITLAILSIITSLAAQDWNSPQLKSFSSAQQECAVYLLLSNETVQQCEVNGYPDDFNCRKLVN
CILVQIYAYDERIGIRDNVITNFFEPPKSCSDYVSRTQECLQTTVPKQCGGQPFERAYLSFQCYYRNYGVL
LKDAVRFLPYDPSSPKYSVKQIVESFSIANTSCKAIRNLSEGRGFTVENVADALYAFGIRNGFYDLQHGL
YVDRLYTQVGVPNLLSEATRQCLACVSQQYNTEPLRITQLVLQCVEKDIATQLWFTQTAQAILASNNS
YCNVCEPLRSCVDPTPSTQCGVSLVTTPKNPYPSI
>AaegOBP95
MRCCLIFMLPLVALQSSLALEHLETSRWKSFREAELESAEYLFITNETLERYRSNGYPDEPSVRKLIGAIM
VVLNAAFEKLNLIKDYVISKYFIPNTVDCLYKQHTKECLDRNVATLDPSDRLGRAYQTFQCYYKNYGG
IKVDVDWVPYHYSEVVQIVEDCLYITNASNESLHQYCRGEYATNAGYQNVVYCYFVRNGFYDKSTGF
NVQRIYNQLGANNLIDDGTEKCITQVVNHHCKEPFRSMRVFLDCVVRYVPSSAAITEAASNILGNPPE
CVVPPSPPPKTQPCYNELCP
>AaegOBP96
MFRIGLLFVSFAVVSITAVDRHKIVYKSLQEAAVECGQYTIKGQCLGRCETLITGDWNDTTGMSPAYSRF

FHPDPVDECNLNRTQRCLQTKVYTVPRPRTCQRASESIQCYLDQFGQVNLTAPQFVRFTPLQDDQIVL
ECAAIMGYTYEQVYAWIRESAFQRPETRCIYRCFLIRSGLYSDSEGLNMARFYVLCGGYEEDFYQRVEQ
CAARLRQEVPCNDKCTLAQRLAIECIGADYQAGNLATNANSKAVEGSRVQNINANPVNSVIDATNSE
TGNVITITRTNSDTYVYGDENTFENYFYESA
>AaegOBP97
MFRIGLFFASFAVVSITAVDRHKIVYKSLQEAAVECGQYTIKGQCLGRCETLITRDWNDTTGMSPAYSRF
FHPDPVDECNLNRTQRCLQTKVYTVPRPRTCQRASESIQCYLDQFGQVNLTAPQFVRFTPLQDDQIVL
ECAAIMGYTYEQVYAWIRESAFQRPETRCIYRCFLIRSGLYSDSEGLNMARFYVLCGGYEEDFYQRVEQ
CAARLRQEVPCNDKCTLAQRLAIECIGADYQAGNLATNVNSKAVEGSRVQSFD
>AaegOBP98
MRNTIICIVFTLCAISPSNVLGLDHYIGYKSFDTYFRECGEYFEVPNCTLDEYAVNAYPDEPEVRNLIHCT
LVGSRSWHDGSGVIESVMANFFNPGPEDTCYADRTRECILSSQVPCDSNITLAYKAFQCYYRQYGNLN
ESSQYMPCTDRELQVLINTSIIMVNVPKDELVNYSNGVQLNQPHFAELLYVIFIRGGFYYTDDKTLALN
NLYTQFGNPELQTPETQQCVNSATAAWDGKRQRDLVYAYFVNCLQKIVPWLQLIQQVATSLSVRPFTP
LAKPYSTTMRIITTGLIFTLCAISASLAQNNLQHYVVYKNFDTYFRECGEYFEVPNCTLDEYVVNAYPD
EPEVRKLIHCALVSFGGWEGGIGVVEYVMSNFFNPGPEDTCYADRTRECIQNSQQPCDSNVTLAYKAF
QCYYRQYGNLNQCLQFIPNSARELQVLIEASIAAVNVPNDELVNYSNGVELDQPHFAELIYVIFLRGGY
YYPGQGLLLKNLYTQFGHPELMTPETQQCVDAATAAWDGKNQRDLVYAYLVNCLQKITPWLQLIQQ
VATSLVTVPPPPCPPPATPCTTTTTTTTTTPPPPNFSP
>AaegOBP99
MMLTGFSSCTPQNGRNGITRGCQRDSLRWSWKKKTKFMWVSSTLLVLSVLCVSAVELPPPHYVTRISF
YTALQECAEYFQISENLLQQYISSSYPDDPSVHKLVRCSLMLLGCWDDITGMRRNVIENFFEIDPNDRD
HVRRTNECIRKSTTEDVSSPAYVAFLCYHQQFGNFKLHSKRFVPFGSHELKQLVEMALNVAELPWFVP
AQYATNDILYEPHFPPVLYFIFVRGGFYNAKIGFDLRNLFTQFGVEELLKADVEQCLANVVHTEKVNG
HESIVIKGFQKCLAHFIPLLEVVQDVARSTSNDRSASVKACTGLNPSTQPPFYNRACED
>AaegOBP100
MGLKKWCIILPYLCSFAICVSALEHVATLKSFDEIRYECSQYLPSSEDEDCSLRCLGLVGRFWNDTIGTPS
NSVGRFYRPDSCDQCYLNRTEQCLRRTVLNLPRSAVCQRASNGLLCYKDQYGQLINRAPQFVPVVKL
RAMRIFRECAQMLEIPFDKVDRIFKEGRNNTSEGRCLTRCFLIRAGLYSDSRGPDIGRFAVQCEGYSVEY
ERTLVQCYEGLKAQQLDSCTLATRVLDECIQNNKYSYSNMDDVVTIQITDFTKLQVMVDLGTLVVFFP
SL
>AaegOBP101
MEETDSNLITWHRVYLHREYVHGIGSETRNSLFHLHNARDLVLVTTCRHGKSWRDLQDARCDEDNR
EYDRTVALKAPTMQLRSKCLVLPLLGLCIFANRAVTLQHTATLKSFDELRIECSRYLPPVDALNNVEDC
SDRCLGLVGRFWNDSISRTVYSVSRFYQPDSCDQDNLDRTEQCLCETVQSLPRNASCQRASCSMQCYQ
DQFGELINQKPQFVPVSKLRSAQIMSDCAQVLQISQDTVRQILHDGYNNTCEGRCLVRCYLIRAGLYS
DRRGPNIARFSVQCEGYADEYERSVTDCYAGLKAQQLDKCTLAARFYDECILSNEYSNSNMDVIAAL
GGSLYGVILTTVGVTGYLVTSIVAGLSAAGIP
>AaegOBP102
MGLKKWCIILPYLCSFAICVSALEHVATLKSFDEIRYECSQYLPSSEDEDCSLRCLGLVGRFWNDTIGTPS
NSVGRFYRPDSCDQCYLNRTEQCLRRTVLNLPGSAVCQRASNGLLCYKDQYGQLINRAPQFVPVVKL
RAMRIFRECAQMLEIPFDKVDRIFKEGRNNTSEGRCLTRCFLIRAGLYSDSRGPDIGRFAVQCEGYSVEY
ERTLVQCYEGLKAQQLDSCTLATRVLDECIQNNEYSFSDLFDLLEPYLDGVISIGDFRRLELMIQLDNLV
IYYPSLPSSSLGI

>AaegOBP103
MQVFAAIVKLVAITLGAIIASISCIEEHSASLKSILSSSAECNLYLPTEALRQECGTRCVSLVNRIWNDTNG
RLSDTIGRFYVEGPQDPCARNRTLQCLEQVTASIPLRNSCQLADASVNCYRNNYGQLDVKSPRFVAFS
DVQQVRILTECAAMLGVWDKLTQVVRNGLQSISEGACLLRCLLIRQGLYSDQRGPDLKRVSVQCGGY
EGYEQEWRANVTRCVAAVRAERICDKCLQAERIAVDCLQMHLHLYEVRSPKLRQHIPFGVEFYTGAN
AAAGSAAAAQAQVTTYITVYYYYWY
>AaegOBP104
MQLFLTLLIFTLCTSAYAFLDHYVGHKRFDTIFRECGVYFQVPNCILDEYVANAFPDEPEVRNLIHCTLV
GSKSWHDGSGVVEHVISNFFNPGPEDTCYADRTRDCIRNSRVPGGNNVTLAYKAFHCYYRQYGNLN
HSEQFMPCSPQELQVLIKTSIAIVNVSQAELVNYSNGAVLDQPNFAELIYVIILRGGFYFTGQGLFLANL
HTQFGNPELLTPETQQCVDAATAAWNGQRQKDLVHAYFVNCLRRITPWMQLIQDVATGLVRGSNAP
CSTSSTTTSTTPSAVQPCYNVGN
>AaegOBP105
MWISAACLILALSFTSTSHVLGLDHYFSYKEFDSYFHECGEYFEVPNCTLDEYTANAFPDDPEVRRLIH
CTMVIFKGWQDGLGVVESVMSDFFNPAPEDTCYADRTRDCIQNSQAPCDSNSTLAYKAFQCYFRQYG
NLNQSRQFMPYTLREEQVLIETAIAIVNVPKDELVNYSNGILLDQPHFADVIYVVFVRGGFYDVVQGLS
LDNLYTQCGKPELLTAETQQCVDAATSAWDGKSRKDLVYAYFVNCLQNVITFAQRIQEVATYLVAVPP
SPCPPAPSTPCPTTTTTTTTTPPPPSTVPPCYNVRN
>AaegOBP106
MISIVSSEDAQPHLFLITQSIRSFPSALAECAQYYELSNCSLNRIVQESYPNEPDVRRLIRCALINVRSWN
DTTGVQEQVMNSYFNPTPEDTCYLNRTRDCIERSRQLPGGDRDVQTRAYDAFICYYRQYGNLNETEQ
FLPFTDEESDQLMISVLSITEVSQEALVQFSEGNILDNKEFPAVLYTLYVRVGFYQDRIVPQHLYIQFGNP
ELLSPQTEQCIEAAVNSLPCEADDKDQVYRIFRNCLVGITRTLELTQSVSRQLLGLEPFCGNGDSGSSTN
APCAITASPASTSQAPYYNTVPR
>AaegOBP107
SIELSNRPSTIMNRIVLLVLISLCSASTVLADGLPHYIAENSFDISLRVCAEYFLVSNETIDGYYQQGFPEIE
EVKQLLRCAMINLGAYDDTFGPLEYVLGNVFKPCPSDTEYAERTRSCVKKALDSICPSDVFSRAYASFM
CYYRGYGNLITDEFFIPNSLLELTQMMLFVQSSLNLPDEVLVQYSQGNILNEPNFPNVLYVWAVRGGYF
SVDEGIQLENLYIQYGIPGLLSQETRQCAADVAQANCNLDLVTLLYNMYVTCLRPLLPFESFVQTFAVE
QLKCKTCGAVQPAKPSYTY
>AaegOBP108
MGLATTSIALLVLSLAAVGRCDLPQYSVYKSLFTALYECGEYLQVDNVTLDQYIYYGYPSIPEVKRLIHC
AMVNVGAWNDNIGVRPNVFRYFFKPNELDTEYEERTQQCLAQICPNEYDQNYRAFETFSCYYRQYG
RLVKEDVFNPLETLEFLQLLQFIKLVLNIPNEKVVQFAAGDYLNDPLFKQALYIGVVRIGALSRDKGFLP
DVGYAQYGYPQLISPCVQKCIADVAAQYMNADKRELVYQVYVQCWYSFLDPFLRSQFQAALDGSLC
DVQVKY
>AaegOBP109
MGLATTSIALLVLSLAAVGRCDLPQYSVYKSLFTALYECGEYLQVDNVTLDQYIYYGYPSIPEVKRLIHC
AMVNVGAWNDNIGVRPNVFRYFFKPNELDTEYEERTQQCLAQICPNEYDQNYRAFETFSCYYRQYG
RLVKEDVFNPLETLEFLQLLQFIKLVLNIPNEKVVQFAAGDYLNDPLFKQALYIGVVRIGALSRDKGFLP
DVGYAQYGYPQLISPCVQKCIADVAAQYMNADKRELVYQVYVQCWYSFLDPFLRSQFQAALDGSLC
DVQVKY
>AaegOBP110
MLSITFALVLFLSASSAVIVSPLDHAIETCGRDLQVCESKLASYRALSFPDDRETQCFIKCVLIELQAWSN

PRRLLKHSTIQQYFIPDAADYSFEDRTRRCLDQTLPNCIPGDSCSRAYWTFLCYKDNYGNLIRQPNQFIP
PTELDIAQHQLDCADILRIPREELLNAETLTNGSNCYARCILLRSEVYSDESGLDLDRLYVLLGYDTEKQ
AFIQYAQQFLATDSANCQTDRCLAAQVPYQLFHELLVKLFKRDGTRDISYVFDVNNV
>AaegOBP111
MSASAWIRVTFLLVVGLVAYCQAQDKYSQMYRGPVVDCREIFGYLNNLKEFFKDECGQLDEGCPWKL
QKAIKWYGRVAKQIKQYVSATNRHSARFKSSRSTDSECYRYLNVLDGNCSVRCRGLVDRLWDDQSGL
GLSITQFYKPDPEDKCYLNRTVRCLKGVSATESCSRVDKYVQCFNDQYGRKDTETARFIPFTTVQHTRI
LMECAAIQGIPVESLQRAAENGSGLPQEACLLRCFLIRQGLYSDAGGLDLERLEVQCGGYGSGWDPV
AVRQCIAKVEDCDKCSKVQRIAKECLQAHFKVLPNPNSDTVESVPFLVEFYVGISVLGINFKGMISGCL
LSIFCFFA
>AaegOBP112
MVQFVILLITHLAQLVTAADRHKIVYKSLQEANNECSLYNVPGGCLPRCVTQITRDWNDTVGMSPVY
GRFFQPDPNDLCSNNRTERCLESKSSLISSKKTCLQASESVQCFMDHYGEINMTAPQFVRFTKLQDVQ
LIFECAAMLGYSSMEQLDALLRDSEFKRQETRCVFRCVMIRSGLYSDSEGLNMPRYYVLCGGYEDGFY
QQAAECSARLRKEVPCDDKCTLAQRMANECIGVDYETSIMQSKGNTVNTIYAIQGSEVYNIDGQNAN
SNVALTSVQRDKTINIENTNSDLNKFGDTINVDNQP
>AaegOBP113
MFTVKYLLTLTSITIVSCSTSEHGFIFKRFHRGLLECAEILNIPKTTVQKSIEDQFRCNDQTKLLIHCVMV
QLHTWSDGTGLRRSALVQFFEPTAYEALFEPRTDMCLTENLAYVDKCDFVTRAYVTFDCFYKQYGNL
ARNVHAVILNQKQLISALNVCFAIADIPQEAIQRLTVENVLEVPEAHCLLYIFSLRAGLYNEVGGVLMD
SIYSQFGNRTLTQSGKITCVQHLLETSRFADRCSMLNAVYDRCLFDAIPINELIVEAAKHALANVGR
>AaegOBP114
MNFDLTLFSIVVLLAISTANATRSPSLKTIDQAVKECGTLWNVSPDYFEDFVRTGTGNSTQLKELVRCAS
IWCRWCNVSAHDVVYEVLQNYFNPSPDDPCFLNRTERCMKASLKDLPYTEVLERAFVSFLCYYQQYG
NLNRSVQFIPYMLPQEQQVALDTLVIHSVPLETLRNFNDGVFKEGTFEFLLRTLLVRLNLYSDRAGPDV
KRLYNQDGNESYLTPETAACIAEARKNCPSDRCKLVSNTLKNCLPQVYDDAVSLIKDAARMILQRMF
CVQDLELNPILVERLVAKGAEEIFTNRHDCVI
>AgamOBP1
MKLVTFVFAALLCCSMTLGDTTPRRDAEYPPPELLEALKPLHDICLGKTGVTEEAIKKFSDEEIHEDEKL
KCYMNCLFHEAKVVDDNGDVHLEKLHDSLPSSMHDIAMHMGKRCLYPEGETLCDKAFWLHKCWK
QSDPKHYFLV
>AgamOBP2
MLAQASPLLLLLLLLVTQCLDGANCSTITTQRPAPRRDGQYPPPETLAFLRPLGKLCLEETGVSPEAIKR
FSDADPFDDNRALKCYMDCMFRVTNVTDDRGELHMGKLLEHVPTEFEDIALRMGVRCTRPKGKDV
CERAFWFHKCWKTSDPVHYYLV
>AgamOBP3
MGHDSCWSSRWRVLAALVIFQCAILMVRSDEPRRDANYPPPELLEKMKPMHDACVAETGASEDAIK
RFSDQEIHEDDKLKCYMNCLFHQAGVVNDKGEFHYVKIQDFLPESMHLITLNWFKRCLYPEGENGCE
KAFWLNKCWKTRDPVHYFLP
>AgamOBP4
MSVSVLVSSLVVLFCVQCLIEHIDGAMTMKQLTNSMDMMRQACAPKFKVEEAELHGLRKSIFPANPD
KELKCYAMCIAQMAGTMTKKGEISFSKTMAQIEAMLPPEMKTMAKEALTHCKDTQTSYKDPCDKAY
FSAKCAADFTPDTFMFP
>AgamOBP5

MAASRSCWWWRWWWDFILGLVAFFFIPFPSVECAMTRKQLINSMDMMRSACAPKFKVSTEMLDNL
RGGIFAEDRELKCYTMCIAQMAGTMNKKGEINVPKTLAQMDAMLPPDMRDKAKEAIHSCRDVQGR
YKDSCDKTFYSTKCLAEYDRDVFLFP
>AgamOBP6
MTSNAFYSSNTVTWVVAVIGVYCLVFRPALVHAQQSLTQADMDEIAKGMRKVCMSRHKISEEMANY
PSQGIFPDDQEFKCYVACLMDLTQTSKKGKLNYDAAVKQIDILPENYRQPFRLGLDSCRTAADDATDR
CEVAYILLKCFFKASPKFFFP
>AgamOBP7
MCEYSNTRNKMSNLVVVLVLLTMYIVLSAPFEIPDRYKKPAKMLHEICIAESGASEEQLRTCLDGTVPT
APAAKCYIHCLFDKIDVVDEATGRILLDRLLYIIPDDVKAAVDHLTRECSHIVTPDKCETAYETVKCYFN
AHDEVIKFCHLLVLE
>AgamOBP8
MPSRKRLCRLLLLLLLPVDLELISQDADANVFPAYPVLRNSTPFSIFQTHGAYVVRTFADATAYRDECV
QQYAGRGSSLIDYMRQVALHTDNADSRWCIVRCILQKADLLDGEGAPHEANVHAQMQHSNAIVED
PDDIRSETSRCLREPPAPDSGGGCLRAYTFFACIQSTEYDLF
>AgamOBP9
MLKFVVALLAFTAVVSAEFVVQTREDLLAYRAECVKSLGVSDELVEKYKSWNFPEDDTTQCYIKCIFNK
MQLFDDTNGPIVDNLVVQLAHGRDANEVREEIVKCAGSNTDGNVCHWAFRGFQCFQKNNLSLIKAS
VKKD
>AgamOBP10
MVRVLIVFVALLTFAGQPFAVRGQQELSDLPEVKGYKLHCIESSGITESSAKKLAAGESIKEPDQPTKCF
VQCFFQKLRLMDEKGVVLKDKLEVFLTKLMDADKAKDYVQQCDLRRTNPCDTAYAVYDCYLGKKA
KLF
>AgamOBP11
MIKPFVCILIVAAGCANAFMYKHPYNHHQAAVLAHEPVVPVEFVKHTTSPAFRPASFLEVMEVVLDC
FNTLRIPLQRFPSYLSGIFPEDPETKCFLRCVAIKLGVYCDEKGADLDRHCVQFGLGECCENFSNRHLV
CLQQNSLPCPDRCTAAYKQELCFQEPIAKYLDYHFHDLVGLLHQAKCSHDLKMLHP
>AgamOBP12
MAPVRYHFVLWLLILIGVSSLVPPGECLDISKVTLDAAFYPLFGCARDLVVPEDLIELYKKRIFPDDQLTC
CVFRCLGMRLGIYDDVKGFDVDKQYERVKDRLSVDEDTYKRGVKNCIRNVLRGRTLNNCEKAYLILN
QCQGNTITNSLNQQLNEIRCN
>AgamOBP13
MKSFQIATLTVLLVLLAGTASAKKASTIFGMPLQQDPVPATSTFIVSDFLQFLQTAVTCFNKLRIPEERFP
LYLAGVFPNCPETQCFVRCLSANLNLYCDETGSDIDRHYLQYGLGQDYNCFRQKAEQCLAANTSPCN
DPCEAAYKQELCFLDEFRKYVDSNMNSLIAAVAVEKAEQNPVYYNMLAHN
>AgamOBP14
MKLSSAVLYFALLATAMVCRVQAGSAEELEQAKEMLRGLAAECKTKEGATDEDVEGFVNDKMPESR
TQKCLAGCMQEQFGVSNGKAFQEDGFIEIAKMLMKGDETKIELAKEIAADCKAVANDDRCELAVDI
MNCLKESAEKHGIELKH
>AgamOBP15/16
MLTIVVATSICLMATASANAPKSLSPELLQQMGQFRSECLRETGTTDEQIEQFNSPQSVQASHELQCYM
YCMFRLHNVTRPNGELDLIDVYHAIPKQFNSIALKVLAKCNKSTGPIADACERAYSHHRCWKETEPEL
RLPVAVCLMF
>AgamOBP18/24

MKIELFTLSAPTVPRPGGPHTEGGRNADNFKLYSSLFVFPSPLQGARLEAEHVRRIHQNARECVKETGI
LPKNAFRVLSGDFSVDTMKAKCFVKCFLDKAGFIDDDGVIQQDVIREKLTVGIEAGKVNELIKKCSVE
GTDACDTAYQMYKCFFSNHKVPKELFQMRKGIGRRNMQQ

>AgamOBP19
MAAYLISVVNYSNYGMYITQEQLEKTARTFRQVCQPKHKISDEVADAVNRGVFADTKDFKCYVSCLL
DIMQVARKGKVNYEKSLKQIDTMLPDHMKPAFRAGLEACKSAAQGVKDHCEAATILLQCFYKNNPK
FVFP

>AgamOBP20
MLFVFFTLLSCTKKKKIFPLRKSTVEQMMKSGEMIRSVCLGKTKVAEELVNGLRESKFADVKELKCYVN
CVMEMMQTMKKGKLNYDASVKQIDTIMPDELAGPMRAALDICRTVADGIKNNCDAAYVLLQCLSK
NNPKFIFP

>AgamOBP21
MQSLQIVFVVLLAAVSTMEQHEIAKSLAEQCRAELGGELPEDFATKMRLGDLTLDSETAKCTIQCMFA
KVGFTLESGAANRDVLIAKLSKGNPTAKAEAFADVCENNEGETACDKAFSLYQCYHKNKSIFD

>AgamOBP22
MNSLLLIGGVLVVLNVQFVTAADNNESVIESCSNAVQGAANDELKVHYRANEFPDDPVTHCFVRCIG
LELNLYDDKYGVDLQANWENLGNSDDADEEFVAKHRACLEAKNLETIEDLCERAYSAFQCLREDYE
MYQNNNNATSE

>AgamOBP23
MKSFFCVASFFLLVASVHAFTLRQQKMVSIFALECMAETGIGAESLTKLRDGDLTANDRTAKCFMKCFF
EKENFMDAEGKLQLEAIATALEKDYERAKIDEMLEKCGEQKEDACETAFNAYACYHDHYQNL

>AgamOBP25
MKFLVFAIVLSAICLDALVDGAAAPPPDLEDVSKIANGEAFALECLIESGLKLDSLAALSAKELDTNGS
KIKCLVKCFFEKTGFMNKDGQLQEETITEQLSKFMPRERIESLVKNCNFQEADACETAYKVTECYFQNK
AGLF

>AgamOBP26
MKTFVAIAVVALIAGTFALTIDQKKKAEGYAAECVKTTGVPPETAAKLKGGDFAGADDKTKCFAKCFL
EKAGFMTDKGEIDEKTVIEKLSVDHDRAKVEGLVKKCNHKEANPCETAFKAYQCIYAAKGAVV

>AgamOBP27
MGRLDLVCLLAIVLLVHSCVSIAWSFSWACTMVKPFVFLLYRMDRTSWDHTSGVAMKPSCFGECFVKR
AGFMNDNFTFNRDTIMRFTNRFVSKEISEKVYNICTDNVTPTYCVTAFDVYQCIYENVYKSWDSRK

>AgamOBP28
MKLLFATVLLAVCAAAQPLTDDQMKKAEGFALGCLEQHKGLNKEHLVLLRDGDFSKVDADTKCFLR
CFLQQANFMDAAGKLQNDYVIERLSLNREKSKVEALVKKCSAGVEVEDSCETAFRAVECYHREKASL
L

>AgamOBP29
MDENTPQKRCVSRAVTVGICGAIVLLLLVGTSPAPVEGLRCRTGEGPSADDVKRIVRTCMNKITNAGG
GNFSSSSSSSTIERDRACLMQCFFEEMKATNADGFPEKHKVLHVITKDIREHELREFYVDSIQECFHML
GLDNRLKDKCDYSMRFVTCLSDRFETNCDDWESVTSAMF

>AgamOBP30
MVTQLSQPLPLRGQHTMATVNLYYLGLVCLLAVTATAASQCFRDAGQLKRVVQAQEECVRYLRIPCA
RLAVYNKFIYPNDAETQCMVRCMGLNLGWWNDTHGVQEASMRSFFHPDPNDCDYERRTYRCLHS
QRLDRPAPHDEACERAYESFRCYYEHYGNLVVTPQFVRLNALQQLDVLLQCADMLQYPMPDRSFSC
AKTHVAGAEGDFDCVLRCYMLRTGLYSEQYGPNLDRIYVQCNNYANETVFRETTDACYQRLRSDCQ

DECTLIARYVRECFPAGGIIFLNSLW
>AgamOBP31
MKQLVLLTICVLALMPLEVLSNDTKGLTIEKSFLQSVHDCAEYLQVPKHRLVQYLAYEFPPDEETKCLIF
CVGTDLRWWNNTCGLQVPAIMNYFQPVLGDRQYEKRTSECLERNVHTAELPNNCCQAYETFQCYFR
EFGNLVTCPQYVPATKLQATQAALDCLTVLRVPTDLLQCYSKGDLPDVPETRCLYHCIDHRTGLYTTES
GIHLSRFYVRDLEVNDLRYLSKETKACRDRVRMSGCDVCSEVYNTHRDCLSGIGVDGYTSGIIAEASRI
ALTNLATALSALPARSYAQRSPYPSFHRTCKAEHFGRSF
>AgamOBP32
MISIELKYITLACVLAATVTAGSHCHNDYYQLKSVSQAQEECARYQGIPCARLAVYNKYIYPNDTQTQ
CMVRCMGLNLGWWNDTHGVQEPAMRSFFHPDPDDCDYERRTYHCLNSQRLNHPSPHVDVCERAY
ESFRCYYEQYGNIVVTPQFVPLSDLQQVDVLLQCANMLPLTVGRSCAGGSKPSERDVDCLARCFLLRS
GLYSEQHGPHLDRLYVQCNNYANETRFRETTGTCYRRLKSECQDECVLAGRFLRECFYEGGISIVNSLP
ASEASVESAGSLGSGQGSAELGESHQEKVLQTWKDLYDRENLQDLWDRQEL
>AgamOBP33
MATIKLKYITLACVLAATVTAGSHCHNDYYQLKSVSQAQEECARYQGIPCARLAVYNKYIYPNDTQT
QCMVRCMGLNLGWWNDTHGVQEPAMRSFFHPDPDDCDYERRTYHCLNSQRLNHPSPHVDVCERA
YESFRCYYEQYGNIVVTPQFVPLSDLQQVDVLLQCANMLPLTVGRSCAGGSKPSERDVDCLARCFLLR
SGLYSEQHGPHLDRLYVQCNNYANETRFRETTGTCYRRLKSECQDECVLAGRFLRECFYEGGLLGSIPV
LGGLGGLVGGLTGLTGLVPPVTLQLTSPGLAAVTLTLSAPSVMVGALPVPPVMVGTLGGAANVGIL
>AgamOBP34
MQFQLNCVQQATRATMNSFALSVFVLAVGAVSVSASLQHYVVEKSFNQAQAECAEYQGVHDDDLL
RYVKEGYPDVEEVRCLLRCVAFNLRFWNHTTGLQKNMVAGHFVPYPDDFHNVERTEACLAENLYTC
DDDLCTQVYKAFQCYYQYYGALSECPQFVVNSYLEDLQVAYDLFGMLAVSQSTLQSLAGGCFPSGEES
LCFFYSFVTRSGLYSVEDGAKLERLYYQYKEEVFNPNNAQTVACLQNQKKLACKKSTCQQAYDTFQN
CFGESRGLEYLLHTVFVDAAKAFLGQPVCYCNKVKTCPLHKCYGR
>AgamOBP35
MNFFTVSAIALVAIIGSIQAEHSPLPHYFVRKSFPEAQAECAVYLQVPDDRLQRYMREGYPDEPEVHCL
VLCVLENLRAWENGTLHENVLANYFVPATEDCDNAKRTERCLVYLPQECNGEPCVQAYRAFQCYYQ
NYGTLTTCPEYVPSYYGEDLQLAYDLFDMLDVSEDTRRKLAGGCFPSGPESQCFFFAYVTRFGAWSKD
APLLHNLYTQSQEDAFKKDNAETNVCLTNLNKLACHKTRCEHATDVFSQCFGNTDLYKHFLAVFKD
AAMTYTRQ
>AgamOBP36
MNFFTVSAIALVAIIGSIQAEHSPLPHYFVRKSFPEAQAECAVYLQVPDDRLQRYMREGYPDEPEVHCL
VLCVLENLRAWENGTLHENVLANYFVPATEDCDNAKRTERCLVNLPQECNGEPCVQAYRAFQCYYQ
NYGTLTTCPEYVPSYYGEDLQLAYDLFDMLDVSEDTRRKLAGGCFPSGPESQCFFFAYVTRFGAWSKD
APLLHNLYTQSQEDAFKKDNAETNVCLTNLNKLACHKTRCEHATDVFSQCFGNTDLYKHFLAVFKD
AAMTYTRQ
>AgamOBP37
MQFQLNCVQQATRATMNSFALSVFVLAVGAVSVSASLQHYVVEKSFNQAQAECAEYQGVHDDDLL
RYVKEGYPDVEEVRCLLRCVAFNLRFWNHTTGLQKNMVAGHFVPYPDDFHNVERTEACLAENLYTC
DDDLCTQVYKAFQCYYQYYGALSECPQFVVNSYLEDLQVAYDLFGMLAVSQSTLQSLAGGCFPSGEES
LCFFYSFVTRSGLYSVEDGAKLERLYYQYKEEVFNPNNAQTVACLQNQKKLACKKSTCQQAYDTFQN
CFGESRGLEYLLHTVFVDAAKAFLGQPVCYCNKVKTCPLHKCYGR
>AgamOBP38

MLTYRAWLLLALLGAQCALILGAPATGHGYDTKSFAQAYLECLRYLNISRQSLYAYDSAAVPLNCGSN
CLLRCIGLNARWWHDETGLSERALVRFFRQAPADSLLQARACVAELPAPPADSCAGAYWSFRCYSDA
LGELIAHPAYVAPCGQEIRRAVSDCATMLQVEDGQLQTCVRTETFLRQGNGAALLRCVVLRLGLYADS
TGVLCDRVRLLMDADTAEQWTVARAEEAKRCEEDLRALGADTCVVAAHAVELCYGWPAFGELWEV
LKQEYGSSDDALAEESEQVVVRRSCTPWMRPLKMGRNRQKARPRRMRKSSCLKMLNRPNWTWQTG
RSR
>AgamOBP39
MASSGQVVAAAAVLLLMQLQTVTSATFGARDPPPPALREAQAACVKYLGICENRLHQYNNSVYPTD
QDTMCMVRCAGIMVGFWDDCQGLKLDGLANLFPALAANDRVRYQIMSCAEKRIATCPPQDTCARA
YNGFRCFLDAQKGGFGAKDMQPQQSTPPQPFDAQEFIRSLSICAKLQRIPKDRRDLYVQGVFPNDDKT
RSLIRCVGIRTGLYDDEQGPNIALLYSLFGAGQSESEFRRRANLCIDANQPLLEAQDKNAQAYVKLYRC
FADQISALVRANANAMA
>AgamOBP40
MERDRSSSYVAAALLLVCISLASAPRGTEANIFGGKLYQKAQQDCILFMGINPLRLDQYKKFVYPPDRD
TMCLIRCIGISLDFWDDILGFDVDLAEQEFSPLVDATFKKYLAGNITLKLELLDPLDNCARAYYAFRTFR
AQIRQFIGTGTTTMAPSVNFQPLTAVQILDIIVDCAREVNLPPSFLTSLTKGIITDCPEVQCLIRCAAVRTG
LYTDKDGALLANLHRQLDPPGEDLASFSLRQGMCLQRNQQPPTADCCTRAFKQFFTCLRPDFEQFFIR
NRETVMQHFLYKTDQPAEDRQPPWCRTMCWIRSGAIWV
>AgamOBP41
MGYWALGTGLQLLLLILVLGGSELQVKAKGSLILRSFDEMVLECAELMSIVHSKLARIRSGVMLPDEDT
KCLIRCVGISGRFWNDHTGLRKELLARYFVTDPADAYNVNRTETCLQELPALELNAEKCCGLAFESFL
CYYYNYGNLRQDSVFVPLDHLQLQHVTSRCMDVHQITTEQLMSLSAEAMDANDKLHCLVRCIGLQT
GVYSDREGVSIDRLNAQYGEGHCEKEFKTHAVECITKHRELAYGSPCKRAYHLLYKCFENVRNVISAY
ELPDSDGN
>AgamOBP42
MFTTRLLVGALVSLGLTACSFAFTEHGAIVQSIVQAQHECVTYLNLPKHRLYQYLMYNYSNDAKTKQ
MLRCVGLILQWWKSDGTLNEHVLAQYFMPDTSDSDYYNRTYRCIERKAPVDDDLCSRAFETFQCYL
QQYGELLNCPKVVPLSDERLTETMHFCLDVLDIPFSDFEQWTSSSELFLHTEPARCLLRCFTIRAGLYSD
QHGPFADRFKLQFGAPKPDVFDNELEGDYCVARLRREGHDACSLAARSLYECYYFADTLLPTFERILPL
LRLVLHQPEVETAEME
>AgamOBP43
MCSNRSAFGLLLLAWLASVTILGVEAYATPPPTTANCTTVSTFDAALQECVVQLGIAPERLDQEYNLLL
YPADRDTMCLVRCIGVLLRFWNDTTGLREATIRQYYEPAPEDQDYQNRTRSCLAALEPSVTDVCERA
HRSFLCYHQHYGYLRKTDRYVPKTPLEMKQIQQDCVDVYGLDPARLNHYQDGQFPDDPETQCFVRC
VGLRAGLYTDRDGPNIDRMYVQCESCADETLFRAKAGECIAAQRRHKLSKCTAAYRTLYHCFRDDQL
DLYASLTTAAATAAAMTTTTTTKKSTPPNAIPALSVRKPSDRAKLSPDAWQLEIILEGLYNQKY
>AgamOBP44
MKQLVCIVVFALVTPNLIVAECDTKGLIVEKSFLQSVHDCTEYLQIPKERLGQYMANEFPPDDETKCLL
FCVGVDLGWWNNTCGLQVPAIVSYFQPVQGDKQYEKRTKECLERRVGAIDSPNSCCQAYETFQCYFQ
EFGNLVTCPQYVRSTKLQATQAALDCLVMLRYPEKLLKVYASGKVEDSPETRCLYHCIDLRTGLYTQN
GISLPRFFVRDAAYNDLRYLSKETKACRDRIRQSGCDKCSEVYNTHTECLSGLGEKGYTSGIITAAAKIA
LTNLCPAVALSYGGRKPSSTCSKASGTGQVYNLSYPGYKSRMSSCSRCGGRGH
>AgamOBP45
MQRRNASGGGVAVLLTAIMALLPTGCDASLDVPHLTLSKSFSRALQDCMEYLQVPGYRYAEYAANSF

PDDPETKCLLRCVGLNLRWWNDTTGMQTAVIEGFFHPDPLDELYENRTAECLRKELSHADTTDCCCL
AYDSFRCYLQHYGNLVPCARFYPEDETRFVRAAQDCIEFLQIPHKLLKSYSAGSFPDAPETRCLLRCFFL
RTGVFHVDTGFDVERLYTRDYEQPDERYLAQETEARLHKLRGSTGDQCTEVYLAYRDVLGELGRAYY
EYDVLQAAAAKMTVCEVAVEPPAMTTTTTTTTTTPTTATACPSTTEFNYKELNCQNCGRLFISNNGRV
SCCRCMKSSTPFGKFFF
>AgamOBP46
MNPIVGKVFLVLCGSLLVTGAPNTCGKLDLKTDPFTCCTIPKLLDVTIVSSCFEKFPIDKDAADKGAAS
MPKTEVTDCMSECILNSTGIYNRRGDVDEKKLNSVFTDSLPANSPWLNVVRKAIKECTAKADKKDKE
FQKDVADQKKATPKGTQVCNPEASFLVDCIHTTVFSDCPTNLRSTSTECDAIWNFLKNCPFSALRQ
>AgamOBP47
MKHLKAFDEAQNDIKAVQKRLSTSSTILSGIQKNMAHLNLLQIGVLSLIAVGSVFAGNPCLKGPPVPK
NAAECCVTPFLVEPSAFMTCHSKWIGQTKRQMAMEGIPRGCCVAECVMNSTSLYSNGKIDREALTKL
YLASTKSMAPEWNKITLDAIDGCFKMADTIKDEIEAGAKLTPAFEGEQICHPISGTILACMGMTLFAEC
PAKLFTVNDDCNKLKSYHSKCPFL
>AgamOBP48
MGQRQRVVVQLALCFLTFGALLQAGVLAGDNPCAAGPPVDTNPAECCPTPMLVDGTIMMDCYKKY
GEQTKKQLQMDGIPRGCCIAECAMNATNMYADGMLKRDDLSKMFMDAVKDKPEWMSLVRDATN
ACFELAEKKMDEIEAGAKLEPSFEGEKICHPISGTILRCMGMMMFAQCPASVFNVNENCNKLREYGSI
CPMI
>AgamOBP49
MEWNWTFLFRSFLLLTLHLLPQSVADDCIDMDLHSMEVARCCRYEPISTEEVAEKCYQELAPNIPPNSS
DFPVCFIDCSYRQMGYITNEANEIDQSKYGQFLAGFDTAYKIAVERAVAACATVQEDIRRDVANVPSK
CNAFALLFHVCVTQITLKHCPDDRWTASEICGKVRMGVPPCA
>AgamOBP50
MHVALPFSVVGKLTCLSPFLQSIKVASCCQLEAFLTLPTYGNCLQTIAEKYPDALWQGTVCAFDCTYRE
MGILTGVDDINVEQISTNQAGYDQAYQEAIAKAVTACMAQKDKIREEADVVQSECSMFAVKFHACVS
LETMRNCPAERWDSSVLCEKVRSGVTVCPL
>AgamOBP51
MCHRVLSLCGFLLLGLQCGWQTLAEDCMDIKIFVSETLQLFRLDGASPKFTAFSSFLQTTKVASCCQLE
EFLTLKTYGNCLNTMAEKYPNSTLDYLVCGLDCTYREMGILTGVDDINVEQISTNQAVYGEAYQEAIG
KAVDACLAQRDEFREQEKFTKSECGMFALKFQGCIMVESMRNCPAERWDSSVLCEKVRSGVAVCPP
>AgamOBP52
MLFKLFTIPFRCPLFFSKHPKQFPPSKKQSELPYCCQTEPLIPEHVSTKCKEREAANHNPGTELFEVCYQ
QCIYEELEAVDGLEIRVEKLYALAEGFPADYRHAVHLAIDECVKRLRKTRHMFEQMNAQCSLFGFAVD
RCVRLLIYENCPTARWSASVACTKSRQGVPFC
>AgamOBP53
MSFRSISALVILLHLFVICTPMPECISQTQKFEVPHCCQMEELIPRPSRTKCQEKAAIDHNPGFQAYFVV
NCLAQCQLEELEVIDGEELHLEKLYPLTAKFPADYRHAVRQAIDECDAWLQGKKKERRRPDGKAHCP
LIGMEVENCLHRTTFSNCPNSRWKASITCNKVRQGLPFC
>AgamOBP54
MDLKKSVAVVFVSFGWMMLLATAADPDCENLKNRREEMEQCCQVNMIIPLDGAEDCSSSVDETSEP
HDKMMCTLECKLKSLGLLNGDDLVEAKVQEYIDRLEGDWKGTAKTIATECITTITEMKKKIQERDHD
MKCSPVGAFFMMCLMKHTQAKCPEDKWQNTSFCNKMRSGECFPKRGRQ
>AgamOBP55

MLPTGLERTVLWVTVIVLVKVMVKSDAQVCCMVEHTFPQEPYRVCHEQHATPQMDNGTVMCIHQC
YYKAIGMFAADGKVNTDAYIKYRDELDPTLRDAFSYSMVVCAKIIAKRMNNNIAEVNRMRCSPLPYL
FNRCLMEVGIGNCPPERWMNCKHG
>AgamOBP56
MLKLALFVGLVGCVVAYDFQDSFYNEVLMEDLLDNADEPIMFGRFRRSASEVQDDKCKRKYKCCND
ANTENMEKIHEIKKQCFMEVRNKNKADGAYEPVDFFSCERLNKTKMEVICAMECVGRKKEVVNEDG
TLIEPKLMEFVKSNFAADDWQQPLLAGHIETCVKEAKEKAAKMPREAGQCSSETSNFGYCMWRQM
ALACPKDKQVANKRCDRIREKLANNEPLHYYKAELEDM
>AgamOBP57
MGKVLILFVGALVVASVTAGRFERSVFAPRIKRDATMRCCNDGFEKSEVHAKFAEVRTACMEELGLGE
TTHEELIKNREHLNCITECIAKKEGIADENGALLHTDLAKVVLEHMSTIEWKVPLAEGFIQQCFDEVEL
TDGAFVPSDEAKCNPEGFDFVFCLWRQFTLACPEEFRDDSEKCVELRDKLTNKEDVSDLHDDIEAAE
>AgamOBP58
MSLHLFVRTSTTHGINMRSSSVWLIVVCAVTVASANSEELLRGKENCLRHDDFPSPNECCSKPQWINR
YAVRRCRYIHAEVDGSRYERGSCEARCGLFKINMTMTDRIQRVRVYRPRLQTRGIDQGWINVVLKALS
YCKPKVTQLQGRHVRTDEEMEQCEIAEDIFGDCVQAQMFMHCPRATWIESRSCQTMRELLATGCPY
KTLGEVVVLNDEGYVRDDRILEEEYDRPYRGRGRTESPRYDYDDNDGYSRGGQYDQRGGNYPRGTER
NRNGNGYGAGDDGGYVV
>AgamOBP59
MPRLLPEQVIETCRARPLPSVIPGVPDPLPENCIAECALNETGILFNGQFRVEQAVKALSTQVPNDTLT
WQHVIEVASKKCYIITVGDSFYLRDVAKNLISPQCIPSSFRFLQCTFSIVYRDCPDIYWNYQNDRCGQFV
VALNNCHYLFRHIWDI
>AgamOBP60
MLSFVFLASIIVGLVSSQPPAPDASCFQPTAVTAEDCCKIPKPIDNAIMEKCRAENPKPGQMPAPGVPRT
EGCCIVQCAMMETGGFVNNALNTDAIKRSMASTLGADSNFGSLVNGAVDTCARQIQNDPAYSVAPIS
SSPDRAGCSFIPQGFVNCLYTALFKSCPAATWTESSDCQALKTKLDSGCPFFLLMGRGPRN
>AgamOBP61
MNRLVCAFGVIFVVATLELVLAHPGKDVLGCHNGTSITVDECCAIPMLANKTVIEKCKAAHPFKPPQ
NTDDKGPRGHPGECLAECIMKGMGALKNEKVDGPAFRKAIEPVVKANPAFAKLLDDTVKQCHESIN
VDSEFTRYVTKPVCKADAKAFINCVYGTLFEQCPTNVWTQKDGCTQLKDKIKKGCAYFALRKHGGR
RMRPT
>AgamOBP62
MKQRCALAGCEKLLPAVLLLLFALQATVPEGTVAGCSMLNNDNAEQRGAAMLADPATVKQVPEVT
MQDAIAQCNRSFIIQPEYLAELNQTGSFPEETDKIPLCFIRCYLKALGILTEDDKVNKEVALARNWATS
GETVDECLEEMAGSACEQAYFFTRCVMTRALVDGKSKDNK
>AgamOBP63
MKTIACLVLASAFIACAVATISEEQREAARQLAGKCMQQTGASEDDVNRLRSGDTEGADRNTRCFVQ
CFFQGAGFVDQDGSVQTDELTQKLASEYGQEKADELVARCRNNDGPDACERSFRLLQCYMENRASL
MF
>AgamOBP64
MGAFESGLGLLGWVAFGMVLLLAGRGCHAQDFKGAIDHCTKDFEMDMDIVVSLKYGDFTERDPLIE
CFTECLMKKSGFMYDDYTYNKTLIIGFAGRYLEPEGAQAVYDNCIDRFGQTVCVTGFEMYQCIHETAV
SEWVSSNF
>AgamOBP65

MQLAICVWTAVCLQRNIIEGFLVELEAFPSSHQQPPKTSPPVRSCGETFNLTDPRTCCSIPYLLPADVVE
PCLEIPLSPIDLAGESNVCVFWQCRAECALNRTEMLVDGHFQLETAMQQLTNATSEDSTLTKRIQYAIG
ACNELFLNCPPQYWTASDECNQLVRTLNNCPHFLVHTDTF
>AgamOBP66
MATTIARIGSANWAKVLVLLWLVQLATAGEPNPACKTMPTVDKDNEDKCCDVPEMFPNETLNACME
EYQKSSKPPLQKSCEITTCVLKKQSLIKSDNTVDKDKIKSYIKEMVKGSDEWKTLVEKAVLEECLPLMD
KDPSNVLSKLKSSLGDCDPAPALTIACAAAKFYVNCPAKDRTKSPMCDEWRTFLSKCSNSLEDLNAIF
MVLENQKTR
>AgamOBP67
MNPVVCAFGVIFVVVTLELVVAHPGKDVLGCHNGTSITVDECCAIPMLANKTVIEKCKAAHPFKPPQ
NTDDKGPRGHPGECIAECIMKGMGALKNEKVDGPAFRKAIEPVVKANPAFAKLLDDTVKQCHESIN
VDSEFTRYVTKPVCKADAKAFINCVYGTLFEQCPTNVWTQKDGCTQLKDKIKKGCAYFALRKHGGR
RMRPT
>AgamOBP68
MATTIARIGSANWAKLLVLLWLVQLATAGEPNPACKTLPTVDKDNEDKCCDVPEMFPNETLNACME
EHQQSSKPPLQKSCEITTCVLKKQSLIKSDNTVDKDKIKSYIKEMVKGSDEWKTLVEKAVLEECLPLMD
KDPSNVLSKLKSSLGDCDPAPALTIACAAAKFYVNCPAKDRTKSPMCDEWRTFLSKCSNSLEDLNAIF
MVLENQKTR
>AgamOBP79
MDRLLLVLLSSASLLLTVYGIKHHIVTKSWSEAQSDCLQYLRVESPGRYLSHRYRDNQTSKQLIFCIILNL
RIYDPTQNVLRLKAMGQFFNPDKTDTLYVNRTNACLLRVKVPPLVDSSEDSQLYSGVMGTLYEVFRCF
YHCYGNINAIAPKLPPTVLELEKIQQECARMVGVSERLLDGGLQLSSHPRYSKLPRCIMLRSGGSVDYL
THRNNSSRRFKLKKNVENDTL
>AgamOBP80
MRRQYSMWASTVAVIACGSALMLLHPVGADAPKKRCLTKPNVSKKVDMVIHQCQEEIKSSLIEDALK
IFTAEHGQWHDRRKRDEGGLDFSHPTIVSHEDKWIAGCLMQCVYRKNNAIDKNGWPTLDGLVSLYT
DGVNEQGYFMATLRGVDRCLKGTSKKYQIKRNDAAENFEQCEVAFDVFDCISDMITDYCSGQMEDD
H
>AgamOBP82
ICGAIVLLLLVGTSPAPVEGLRCRTGEGPSADDVKRIVRTCMNKITNAGTMGEWGQRDRNGEEQQM
MRDYGRSHRRRKRQYYGGQTSGSSSSGSAGEHSYNGRASPQYGEAGQGGNGTRSGGNSSSSSSSTIER
DRACLMQCFFEEMKATNADGFPEKHKVLHVITKDIREHELREFYVDSIQECFHMLGLDNRLKDKCDY
SMRFVTCLSDRFETNCDDWESVTSAMF
>AgamOBP83
SITQEQLEKTARTFRQVCQPKHKISDEVADAVNRGVFADTKDFKCYVSCLLDIMQVARKGKVNYEKSL
KQIDTMLPDHMKPAFRAGLEACKSAAQGVKDHCEAAAILLQCFYKNNPKFVFP
