# Supplementary material for: Function and Characterization Analysis of BodoOBP8 from Bradysia odoriphaga (Diptera: Sciaridae) in the Recognition of Plant Volatiles and Sex Pheromones
Source: Insects. 2021 Sep 28;12(10):879. doi: 10.3390/insects12100879 (PMC8539145; doi:10.3390/insects12100879)
Supplement: Supplementary file 1 [file insects-12-00879-s001.zip › Table.pdf]

Table 1. Chemical compound in the binding assays.

Chemical compound	Molecular	Formula	Pourity	Source	CAS
Nonane	128.26	C_9H_{20}	98%	Sigma-Aldrich	111-84-2
Dodecane	170.33	$CH_3(CH_2)_{10}CH_3$	98%	Sigma-Aldrich	112-40-3
Tetradecane	198.39	$C_{14}H_{30}$	98%	Sigma-Aldrich	629-59-4
Hexadecane	226.44	$C_{16}H_{34}$	98%	Sigma-Aldrich	544-76-3
Ocimene	136.24	$C_{10}H_{16}$	98%	Sigma-Aldrich	3016-19-1
ß-pinene	136.23	$C_{10}H_{16}$	98%	Sigma-Aldrich	127-91-3
ß-caryophyllene	204.35	$C_{15}H_{24}$	98%	Sigma-Aldrich	87-44-5
(R)-(+)-Limonene	136.23	C_{10}H16	98%	Sigma-Aldrich	5989-27-5
α-Humulene	204.36	$C_{15}H_{24}$	98%	Sigma-Aldrich	6753-98-6
cis-3-Hexen-1-ol	100.16	$C_6H_{12}O$	98%	Sigma-Aldrich	928-96-1
1,8-Cineole	154.25	$C_{10}H_{18}O$	98%	Sigma-Aldrich	470-82-6
Citronellol	156.26	$C_{10}H_{20}O$	98%	Sigma-Aldrich	106-22-9
Linalool	154.25	$C_{10}H_{18}O$	98%	Sigma-Aldrich	78-70-6
Butyl levulinate	172.22	$C_9H_{16}O_3$	98%	Sigma-Aldrich	2052-15-5
Methyl phenylacetate	150.17	$C_9H_{10}O_2$	98%	Sigma-Aldrich	101-41-7
Methyl salicylate	128.17	$C_7H1_2O_2$	98%	Sigma-Aldrich	119-36-8
2-Hexanone	100.16	$C_6H_{12}O$	98%	Sigma-Aldrich	591-78-6
ß-Ionone	192.30	$C_{13}H_{20}O$	98%	Sigma-Aldrich	14901-07-6
Decanal	156.26	$C_{10}H_{20}O$	98%	Sigma-Aldrich	112-31-2
Valeric aldehyde	86.13	$C_5H_{10}O$	98%	Sigma-Aldrich	110-62-3
Octanal	128.21	$C_8H_{16}O$	98%	Sigma-Aldrich	124-13-0
Benzaldehyde	106.12	C_6H_5CHO	98%	Sigma-Aldrich	100-52-7
Dodecyl aldehyde	184.32	$C_{12}H_{24}O$	98%	Sigma-Aldrich	112-54-9
Heptanal	114.19	$C_7H_{14}O$	98%	Sigma-Aldrich	111-71-7
Nonanal	142.24	$C_9H_{18}O$	98%	Sigma-Aldrich	124-19-6
Diallyl disulfide	162.26	$C_6H_{10}OS_2$	98%	Sigma-Aldrich	2179-57-9
Methyl allyl disulfide	120.24	$C_4H_8S_2$	98%	Sigma-Aldrich	2179-58-0
Acetophenone	120.14	C_8H_8O	98%	Sigma-Aldrich	98-86-2
Carvacrol	150.22	$C_{10}H_{14}O$	98%	Sigma-Aldrich	499-75-2
（H-11）Indole	117.15	C_8H_7N	98%	Sigma-Aldrich	120-72-9
n-Heptadecane	240.47	$C_{17}H_{36}$	98%	Sigma-Aldrich	629-78-7

Table 2. Binding data for the recombinant BodoOBP8 with different plant volatiles.

Chemical compound	BodoOBP8	
	**IC50(μ M)**	**Ki(μ M)**
n-Heptadecane	6	4.04
Nonane	>50	>50
Dodecane	>50	>50
Tetradecane	>50	>50
Hexadecane	>50	>50
cis-3-Hexen-1-ol	>50	>50
Citronellol	>50	>50
Linalool	>50	>50
1,8-Cineole	>50	>50
Butyl levulinate	>50	>50
Methyl phenylacetate	>50	>50
Methyl salicylate	>50	>50
Acetophenone	>50	>50
Ocimene	>50	>50
ß-pinene	>50	>50
ß-caryophyllene	>50	>50
(R)-(+)-Limonene	>50	>50
α-Humulene	>50	>50
Decanal	>50	>50
Valeric aldehyde	>50	>50
Octanal	>50	>50
Benzaldehyde	>50	>50
Dodecyl aldehyde	>50	>50
Heptanal	>50	>50
Nonanal	>50	>50
ß-Ionone	>50	>50
2-Hexanone	>50	>50
Carvacrol	>50	>50
（H-11）Indole	>50	>50
Diallyl disulfide	10	6.73
Methyl allyl disulfide	8	4.04
