# Supplementary figures and images for: Function and Characterization Analysis of BodoOBP8 from Bradysia odoriphaga (Diptera: Sciaridae) in the Recognition of Plant Volatiles and Sex Pheromones
Source: Insects. 2021 Sep 28;12(10):879. doi: 10.3390/insects12100879 (PMC8539145; doi:10.3390/insects12100879)

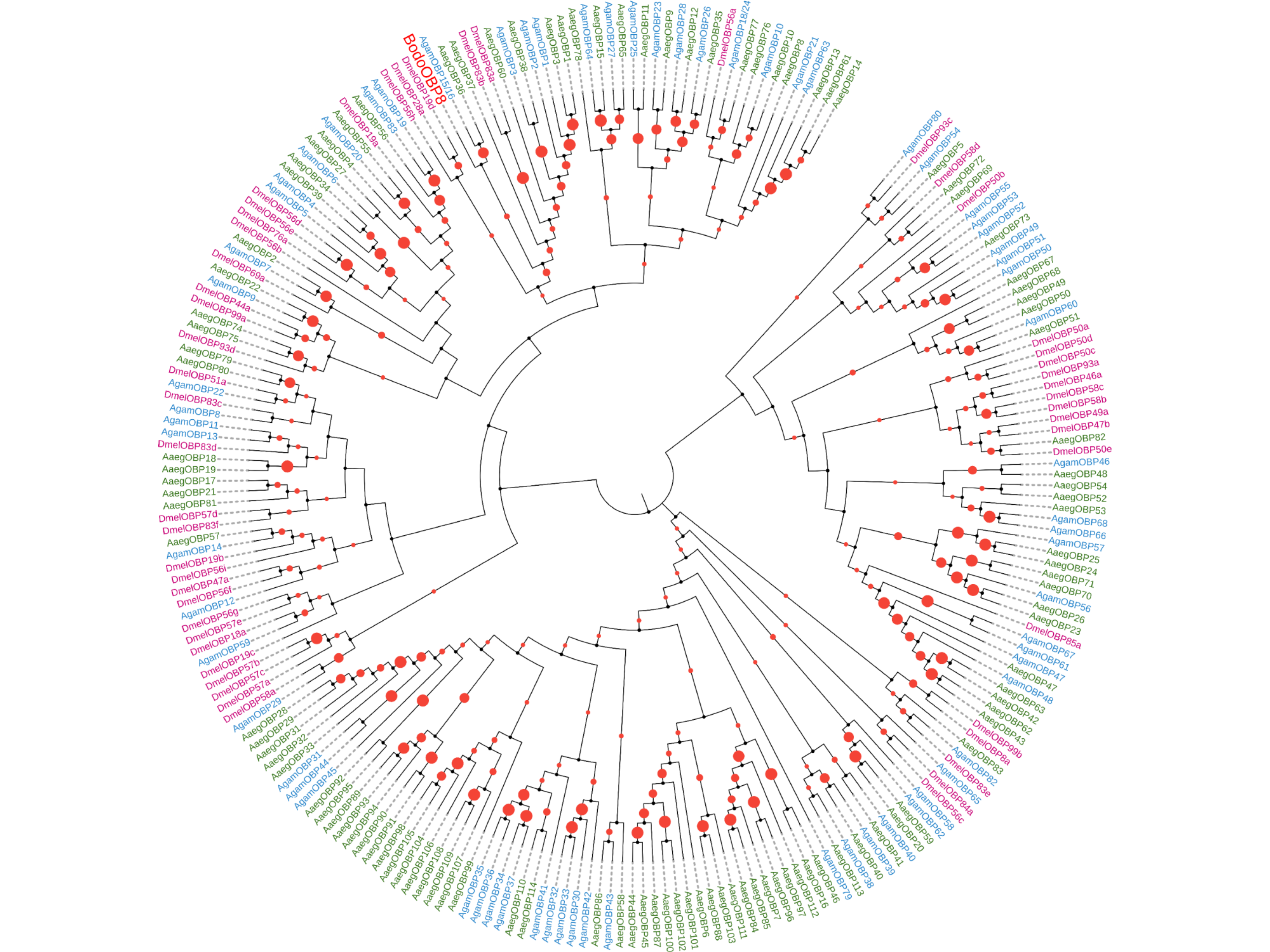

Supplement: Supplementary file 1 [file insects-12-00879-s001.zip › Figure S1.tif]

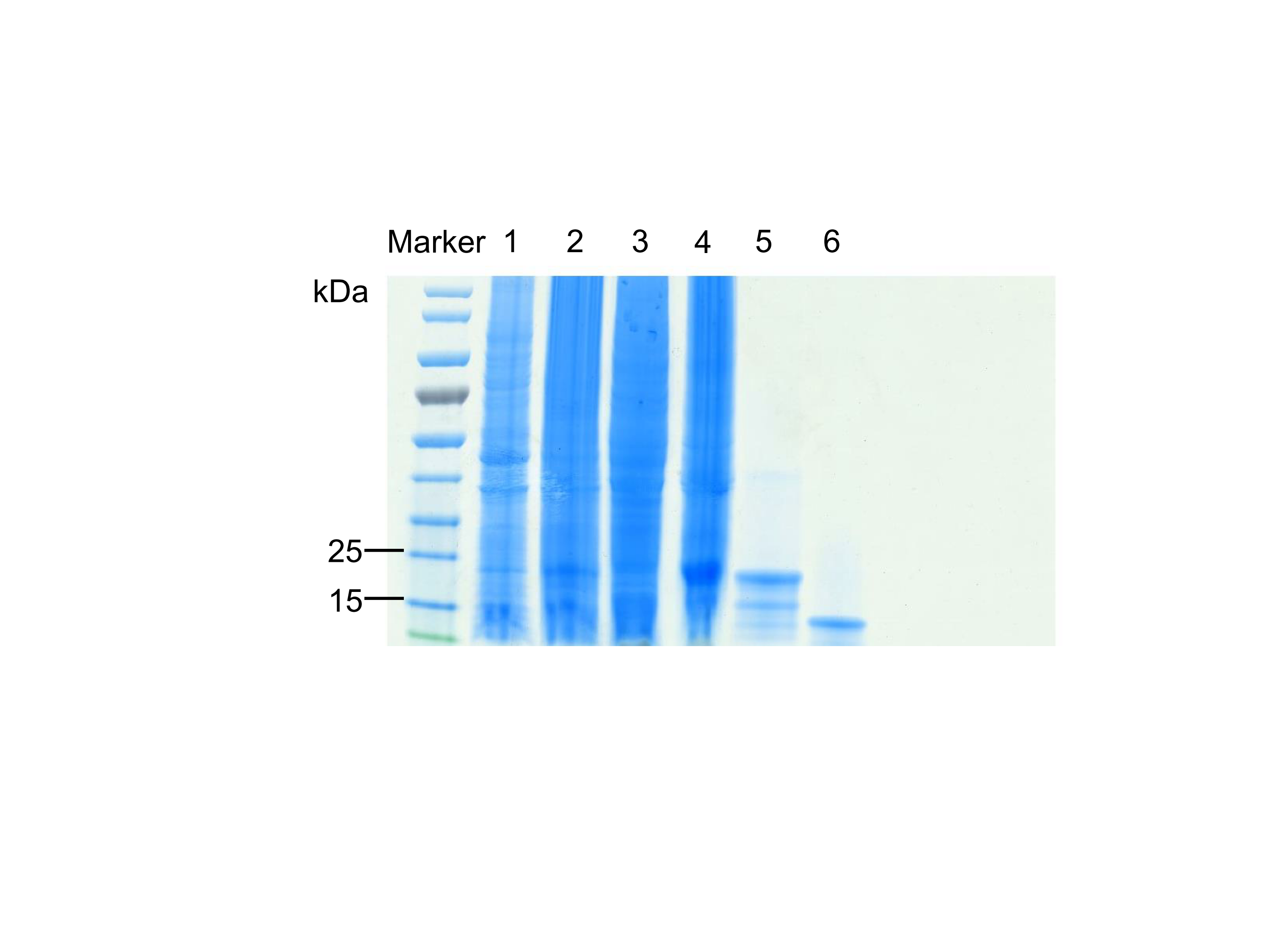

Supplement: Supplementary file 1 [file insects-12-00879-s001.zip › Figure S2.tif]
